# Disrupted bilateral co‐ordination of soleus motor units during early subacute stroke rehabilitation

**DOI:** 10.1113/JP290882

**Published:** 2026-09-18

**Authors:** Jackson T. Levine, Xin S. Yu, Alyssa Jones, Rebecca Muñoz, Martin Zaback, Christopher K. Thompson, Dario Farina, Simon Avrillon, José L. Pons

**Affiliations:** ^1^ McCormick School of Engineering Northwestern University Evanston Illinois USA; ^2^ Shirley Ryan AbilityLab Chicago Illinois USA; ^3^ Department of Physical Medicine and Rehabilitation Evanston Northwestern University Illinois USA; ^4^ Doctor of Physical Therapy Program, College of Health Sciences University of Memphis Jackson Tennessee USA; ^5^ Department of Physical Therapy University of Illinois Chicago Chicago Illinois USA; ^6^ Department of Health and Rehabilitation Sciences Temple University Philadelphia Pennsylvania; ^7^ Department of Bioengineering Imperial College London London UK; ^8^ Movement‐Interactions‐Performance (MIP), UR 4334 Nantes Université Nantes France

**Keywords:** balance, motor units, rehabilitation, stroke, synchronization

## Abstract

**Abstract:**

Stroke can affect sensorimotor control, impairing balance and locomotion. These impairments increase fall risk, limit patient independence and reduce quality of life. Here, we investigated how stroke affects bilateral co‐ordination of soleus motor units during standing in individuals undergoing subacute rehabilitation. Fourteen individuals were recruited after admission for inpatient rehabilitation with sixteen age‐ and sex‐matched controls. Both groups attended two sessions separated by 1 week, during which high‐density electromyography (EMG) signals were recorded from soleus muscles when standing on force plates. Patients also performed the Berg Balance Scale (BBS). EMG signals were decomposed into motor unit spike trains, from which peristimulus time histograms and EMG waveform averages were computed in relation to peaks in centre of pressure (COP). The amplitude of motor unit and EMG activity around COP peaks, directional tuning and bilateral synchronization were estimated. Individuals post‐stroke improved their BBS score between visits, but COP displacement and speed were still greater than controls. Soleus activity was tuned anteriorly in both control and non‐paretic limbs, whereas it was tuned laterally in paretic limbs. Synchronization of soleus activity was also reduced in the paretic limb compared to controls. Furthermore, cross‐correlation lag between limbs was greater in individuals post‐stroke than in controls at visit 1, whereas it decreased to control levels at visit 2 (*P = *0.008). The decrease in lags was strongly correlated with improvement in BBS score (*R = −*0.94, *P *< 0.001). These results highlight the importance of bilateral motor unit co‐ordination in balance, which is disrupted in both spatial and temporal domains following stroke.

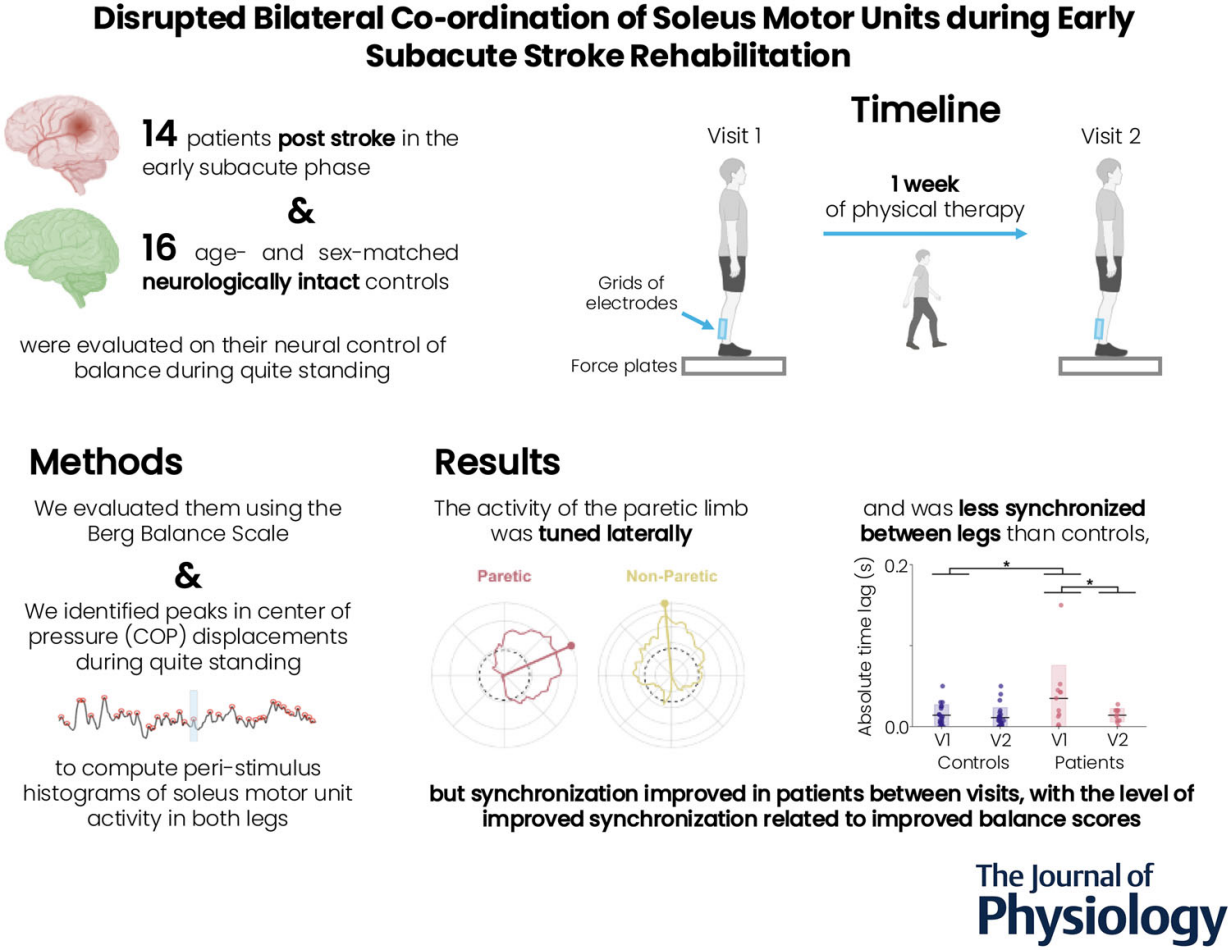

**Key points:**

- We examined bilateral motor unit co‐ordination during quiet standing in individuals in the early subacute phase following unilateral stroke, whereas previous works have focused on the chronic phase post‐stroke.
- Although the non‐paretic limb of patients exhibited a spatial tuning of its activity oriented in the same direction as those of neurologically intact controls, the spatial tuning of the activity of the paretic limb was altered.
- Bilateral synchronization between soleus motor units was reduced in individuals post‐stroke in comparison with neurologically intact controls.
- The level of bilateral synchronization between soleus motor units was associated with balance function and improved after one week of inpatient rehabilitation.

## Graphical abstract

**Abstract figure legend** Fourteen individuals with stroke undergoing inpatient subacute rehabilitation and sixteen age‐ and sex‐matched neurologically intact controls were studied. We assessed Berg Balance Scale scores and motor unit activity during quiet standing, examining activation of the soleus muscle during shifts in centre of pressure before and after 1 week of rehabilitation in the stroke group. Although control and non‐paretic limbs were directionally tuned to activate during anterior shifts in centre of pressure, the paretic limb was directionally tuned to activate during lateral shifts. Interlimb synchronization, quantified as the absolute lag in cross‐correlation, was greater in the stroke group at baseline but decreased to control levels following rehabilitation, with these improvements correlating with gains in balance performance.

## Introduction

Stroke is a neurological deficit resulting from an acute focal injury to the central nervous system caused by a vascular event (Campbell & Khatri, 2020). This injury dramatically impairs cognitive and sensorimotor functions, with, for example, half of stroke survivors exhibiting balance impairments (Khan & Chevidikunnan, 2021). These impairments are characterized by greater sway variability and greater speeds (Dickstein & Abulaffio, 2000; Garland et al., 2003, 2007; Mizrahi et al., 1989), primarily resulting from pathological neural activation of postural muscles and their co‐ordination. Garland et al. (2003; 2007), for example, identified a reduced activation of the soleus muscle in the non‐paretic limb using electromyographic (EMG) recordings, with fewer differences between limbs observed following rehabilitation. Furthermore, investigations into the bilateral co‐ordination during standing balance have revealed reduced synchronization between limbs in individuals post‐stroke (Arya & Pandian, 2014; Dietz & Schrafl‐Altermatt, 2016; Yamanaka et al., 2023).

These changes underlie alterations in the activation and co‐ordination of motor units, comprising the neural structures that convert neural signals into motor outputs. In neurologically intact individuals, motor units that serve the same function receive a high degree of common synaptic input from both supraspinal and spinal circuits (Castronovo et al., 2015; Negro et al., 2016; Thompson et al., 2018), constraining them to co‐ordinated activation and motor output. During unperturbed standing balance tasks in neurologically intact individuals, motor units from the soleus are tonically activated for weight support and show some degree of rate modulation. Motor units from gastrocnemius medialis are recruited intermittently during larger balance adjustments and gastrocnemius lateralis exhibit little activation (Cohen et al., 2023b; Héroux et al., 2014; Hug, Del Vecchio et al., 2021; Pollock et al., 2014; Vieira et al., 2012). Motor unit activation varies with the magnitude and direction of postural sway, with soleus motor units demonstrating the greatest modulation with centre of pressure (COP) movements along the anteroposterior axis (Cohen et al., 2023a; Zaback & Thompson, 2026). Bilateral motor unit synchronization is present during balance tasks in neurologically intact individuals (Boonstra et al., 2009; Gibbs et al., 1995).

In individuals post‐stroke, motor units in the paretic limb exhibit reduced discharge rates, compressed rate coding and altered recruitment patterns, and the extent of these changes are moderately correlated with functional impairments (Beauchamp et al., 2023; Chou et al., 2013; Hu et al., 2015, 2016; Kallenberg & Hermens, 2011; Li et al., 2015; McNulty et al., 2014; Miller, Thompson et al., 2014; Mottram et al., 2014; Negro et al., 2020; Tacca et al., 2026). During standing balance, a reduced correlation between bilateral motor unit discharge rates has been observed in individuals with chronic hemiparetic stroke (Garland et al., 2014), suggesting they may arise from long‐term adaptation in motor unit properties or numbers (Li et al., 2011; Lukács, 2005) or from the reorganization of synaptic inputs to these motor pools (Beauchamp et al., 2023; Karbasforoushan et al., 2019). Whether these changes occur during the acute phase of stroke, or emerge only over time, remains to be determined.

Here, we aimed to compare the activity of motor unit pools during a quiet standing balance task in patients in the early subacute phase post‐stroke and in neurologically intact controls. We conducted two experimental sessions 1 week apart during inpatient rehabilitation. We specifically investigated soleus motor unit activation during COP peaks, focusing on the amplitude and directional tuning, as well as bilateral co‐ordination of motor units. We hypothesized that individuals post‐stroke would exhibit greater differences in directional tuning between legs, particularly in the paretic limb as a result of previously reported weakened neural drive (Kallenberg & Hermens, 2011; McNulty et al., 2014). Accordingly, we also hypothesized that the bilateral co‐ordination of motor units would be reduced in individuals post‐stroke compared to controls. Finally, we hypothesized that motor unit bilateral co‐ordination in individuals post‐stroke would be related to the level of impairments quantified with the Berg Balance Scale (BBS).

## Methods

### Ethical approval and participants

Fourteen individuals with subacute stroke admitted for inpatient rehabilitation were recruited for this study (time since stroke to first session: 19 ± 8 days, *n = *7 females; age = 60.2 ± 15.9 years, height = 171.0 ± 6.1 cm, weight = 77.6 ± 16.3 kg) (Table 1). The sample size for this study was determined based on feasibility and was consistent with prior work performed in healthy humans (14 neurologically intact participants; Zaback & Thompson, 2026). In the present study, we recruited a comparable number of participants post‐stroke (*n* = 14). The sample size is also consistent with previous motor unit studies in chronic stroke populations (Garland et al., 2014; Hu et al., 2015; Hu et al., 2016; Kallenberg & Hermens, 2009; Li et al., 2015; McManus et al., 2017; Mottram et al., 2014; Murphy et al., 2018; Negro et al., 2020).

**Table 1:** Participant demographics

Stroke Participant	Age (years)	Sex	Stroke type	Stroke location	Hemi side	Time stroke to admission (days)	Time stroke to session 1 (days)	Baseline BBS (out of 56)
1	62.0	Male	Ischaemic	MCA	Left	16	24	48
2	75.7	Female	Ischaemic	MCA	Right	6	18	28
3	71.1	Male	Ischaemic	Thalamus	Right	4	11	34
4	56.6	Female	Haemorrhagic	BG	Left	10	15	29
5	19.0	Male	Haemorrhagic	FP	Left	10	15	5
6	75.8	Male	Ischaemic	PCA	Right	4	10	30
7	67.2	Female	Ischaemic	MCA	Right	17	26	37
8	65.8	Male	Ischaemic + Haemorrhagic	MCA	Right	7	35	24
9	58.9	Female	Haemorrhagic	PLIC	Right	5	10	19
10	55.3	Female	Haemorrhagic	BG	Left	20	27	20
11	78.9	Male	Ischaemic	FP	Right	5	12	34
12	55.4	Male	Ischaemic + Haemorrhagic	MCA	Right	12	26	43
13	37.9	Female	Ischaemic	MCA	Right	28	30	38
14	63.1	Female	Ischaemic	PLIC	Left	9	13	9
**Mean**	**60.2**	**M/F**			**R/L**	**10.93**	**19.00**	**28.00**
**SD**	**15.9**	**7/7**			**9/5**	**7**	**8**	**12**

Abbreviations: BG, basal ganglia; FP, fronto‐parietal; MCA, middle cerebral artery; PCA, posterior cerebral artery; PLIC, posterior limb of internal capsule.

Sixteen age‐ and sex‐matched controls (*n = *8 females, age = 61.2 ± 14.7 years) were also recruited. Two additional controls were recruited to increase the likelihood of decomposing sufficient motor units to establish normative levels of motor unit function. Individuals post‐stroke were included upon meeting the following criteria: (1) first stroke (ischaemic or haemorrhagic); (2) supratentorial stroke location; (3) study completion within first 6 weeks post‐stroke; (4) currently admitted to inpatient rehabilitation and enrolled throughout the entirety of the study (at least 1 week from baseline assessment); (5) score of less than or equal to 1 on question 1b (i.e. asking what month it is and the age of the participant) and 0 on question 1c (i.e. asking participant to open/close eyes or grip/release the therapist's hand) of NIH Stroke Scale for cognitive function (Brott et al., 1989); (6) baseline visible muscle contraction of ankle plantarflexors; (7) no history of other neurological condition or injury that would impact motor recovery; and (8) no concurrent participation in other lower limb research studies that could confound results. The institutional review board of Northwestern University approved the protocol of this study (STU00214290), which followed the standards of the *Declaration of Helsinki*. Participants (or a power of attorney) provided their informed written consent before baseline assessment. Consent and baseline assessment were completed in the first session for all participants. The second session replicated the baseline assessment 1 week later. All patients received standard physical therapy throughout the study as dictated by their clinicians. This consisted of a standard of 180 minutes of skilled therapy per day for 5–6 days per week, distributed between physical therapy, occupational therapy and speech therapy.

### Protocol

Participants stood on a split‐belt treadmill (Woodway, Waukesha, WI, USA) instrumented with force plates when standing shoulder‐width apart with both feet aligned anteriorly and eyes open or eyes closed. Each trial lasted 30 s. All participants wore a harness for safety that did not provide support to the participant during standing, and a physical therapist provided assistance as necessary to prevent patients from falling. The level of assistance was recorded for each task by an experienced physical therapist on the following scale: independent (0), supervision (1), touch assistance/supervision (2), touch assistance (3), partial assistance (4) and substantial assistance (5). Ground reaction forces were recorded from the instrumented treadmill at a sampling rate of 1500 Hz using a 16‐bit A‐D converter. Force and EMG data were synchronized using an external trigger (Micro1401; CED, Cambridgem UK) sent to both acquisition systems (EMG‐Quattrocento; 400‐channel EMG amplifier; OT Bioelettronica, Torino, Italy).

The BBS was recorded weekly as a part of standard clinical care and results from the clinical assessments were obtained for this study.

### Electromyographic recording

Surface EMG signals were recorded from the soleus muscle of both limbs using two‐dimensional adhesive grids of 64 electrodes (GR08MM1305, 13 × 5 gold‐coated electrodes with one electrode absent on a corner; interelectrode distance: 8 mm; OT Bioelettronica). The grids were positioned lateral to the Achilles tendon in the muscle shortening direction. The skin was shaved and cleaned with an abrasive pad and an alcohol wipe prior to placing electrodes. A disposable adhesive foam layer held the grid of electrodes on the skin, and its cavities were filled with conductive paste to improve the skin‐electrode contact. Reference electrodes (Ag/AgCl snap electrodes; Noraxon, Scottsdale, AZ, USA) were placed over the patella, and a wetted strap ground electrode was placed around the ankle. The EMG acquisition system recorded signals in a monopolar montage at a sampling rate of 2048 Hz.

### Data analysis

#### Centre of pressure

The COP displacement was computed along the anteroposterior and mediolateral axes from the split‐belt treadmill and low pass filtered with a third order Butterworth filter at 5 Hz. Root mean square (RMS) COP displacement and COP speed were computed for the anteroposterior and mediolateral directions as measures of balance variability and total displacement.

#### Bipolar EMG

EMG signals from each channel were visually inspected offline. Channels with significant artifacts as a result of movements or disrupted electrode‐skin contact were manually excluded from further analyses. Across control and stroke participants, an average of 1.5 and 1.9 channels out of 64 were removed, respectively. Bipolar EMG signals were computed by taking the differential of every pair of artifact‐free channels along the five columns and were then bandpass filtered (second order Butterworth filter, 20–500 Hz cutoff frequency) and rectified. The mean of all bipolar signals across the grid was computed and used for the analyses on EMG amplitude.

#### EMG decomposition

Bandpass filtered monopolar EMG signals were decomposed into motor unit pulse trains using a blind source separation algorithm (Negro et al., 2016). Decomposition was run using the peel‐off option, and a silhouette value of 0.85 was used as a threshold to keep the valid motor units pulse trains. Following the automatic peak detection identifying the discharge times, all motor unit pulse trains were visually inspected and manually edited to remove false positives (artifacts) and negatives (missed peaks) when necessary (Avrillon et al., 2024; Del Vecchio et al., 2020; Hug, Avrillon et al., 2021). Motor units were considered as duplicates when they shared at least 30% of their discharge times with a tolerance of one data point (i.e. 0.5 ms) (Holobar et al., 2014). Motor unit activity and EMG signals were then downsampled to 1500 Hz to maintain temporal alignment with force data. Muscles with less than three identified motor units were excluded from further motor unit analyses.

#### Peristimulus waveforms and directional tuning

Previous work has demonstrated that reversals in anteroposterior COP movement are accompanied by phasic changes in soleus muscle activation (Loram et al., 2005; Zaback et al., 2023). Therefore, by trigger‐averaging motor unit and EMG activation in relation to prominent COP peaks, it is possible to investigate the co‐ordination of phasic neural drive to bilateral motor unit pools in individuals post‐stroke compared to neurologically intact individuals.

Anteroposterior and mediolateral COP prominent peaks were extracted and used to segment the motor unit discharge times and EMG time‐series data. In neurologically intact individuals, the standard deviation of the high‐pass filtered (second order, 0.1 Hz cut‐off dual‐pass Butterworth) COPs was used to set an individualized threshold for identifying prominent peaks. In individuals post‐stroke who often display large balance adjustments that can skew this threshold, standard deviation was calculated over windows of high‐pass filtered COP data (window size = 5 s, step size = 1.25 s, total of 24 segments per 30 s trial) and the lower 25th percentile of these values was used to set an optimal peak detection threshold that would be unaffected by the presence of aberrant fluctuations.

Windows of 3 s centred around the peaks were used to segment motor unit discharge times and EMG activity to analyse motor unit and muscle activations relative to COP peaks. Motor unit peristimulus time histograms (PSTH) were computed relative to COP peaks in both anteroposterior and mediolateral directions. The PSTH was computed over 5 ms bins with all motor unit firings from a muscle pooled together and normalized by the number of peaks in each direction. Only motor units that were discharging repetitively during the pre‐stimulus period were included in this analysis (>3 spikes without any interspike intervals >400 ms). PSTH were baseline corrected for each direction by subtracting the mean values computed between –1500 ms and –1000 ms. Similarly, EMG signals were centred around COP peaks in the anteroposterior and mediolateral directions, and baseline‐corrected for each direction by subtracting the mean values computed between –1500 ms and –1000 ms. To quantify the PSTH and EMG activation amplitude during COP peaks in the four directions, the area under the curve from –400 ms to 0 ms relative to the time of the COP peak was computed. This time was selected to quantify the activity that resulted in the change in direction and the resulting COP peak, as performed in previous works (Zaback & Thompson, 2026; Zaback et al., 2023).

This analysis was then extended to investigate how each motor unit pool activity is tuned with stabilizing torques oriented throughout the entire two‐dimensional transverse plane. Using procedures described by Zaback & Thompson (2026), the two‐dimensional COP data were iteratively rotated in 5° increments, generating a total of 72 unique signals representing the unidimensional displacement of the COP across the transverse plane. The same peak detection algorithm described above was applied to each signal, and these event time stamps were used to generate 72 baseline‐corrected PSTH and waveform‐averaged EMG signals representing motor unit and muscle activation time‐locked to stabilizing adjustment in different directions (Fig. 1). The area under the curve of the PSTH and EMG was estimated between −400 ms and 0 ms relative to the peak for each direction, and directional tuning curves were generated from the area values. The circular mean of each tuning curve (Berens, 2009), which represents the direction of COP displacement generally associated with the greatest muscle activation, was computed. To enable comparison between left and right limbs without introducing side bias, directional tuning curves from the left limb were reflected across the anteroposterior axis. This transformation aligned both limbs to a common co‐ordinate system such that anterior = 0°, posterior = 180°, medial = 270° and lateral = 90° regardless of limb side (Fig. 1). A medial COP peak was defined as a peak directed toward the contralateral limb, whereas a lateral COP peak was defined as a peak directed away from the contralateral limb (i.e. toward the side of the limb of interest).

**Figure 1:**
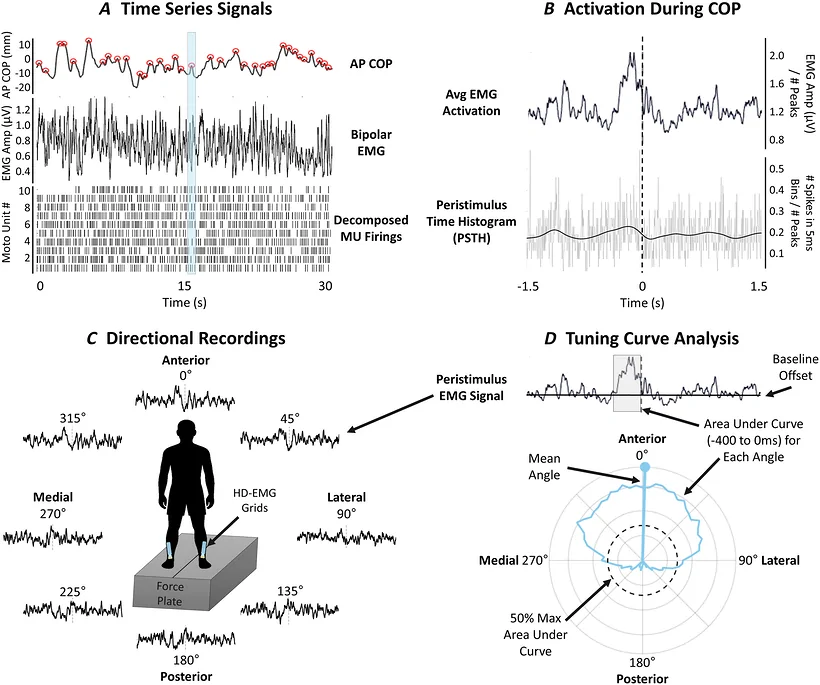
Experimental approach *A*, anteroposterior (AP) centre of pressure (COP), bipolar EMG signal, and decomposed motor unit (MU) firings were extracted during a 30 s trial where participants stood on two force plates during quiet standing, as shown in (*C*). Data were synchronized, peaks in the COP were identified, and, for each COP peak, a 3 s window of EMG and MU activity was extracted around the peak, as shown with the blue transparent rectangle. *B*, EMG and MU peristimulus time histogram (PSTH) waveform averages time‐locked to COP peaks were computed. *C*, time‐locked waveform averages (EMG signals shown in the figure) were computed during COP peaks in the anterior, posterior, medial and lateral directions within the transverse plane of axial rotation, as well as in 5° increments around the participant. *D*, directional tuning curves were constructed across these 72 directions separated by 5° and the circular mean computed to determine the directional tuning of each muscle activity. The dashed line represents the 50^th^ percentile of the area under the curve.

#### Contralateral‐triggered motor unit and EMG activation

To assess bilateral co‐ordinated activation between soleus muscles, phasic bursts of activity were identified from the cumulative spike train and EMG time series separately for each muscle. The timing and amplitude of activity observed in each muscle were then examined relative to the phasic bursts of the contralateral muscle.

Thus, the cumulative spike train of each soleus was first computed by convolving the summed spike trains of all motor units within each leg with a 400 ms Hanning window, and peaks in activity were identified using the same peak detection algorithm as above to identify prominent peaks in COP time‐series data. The 40th percentile of the standard deviation was used as a threshold instead of the 25th, utilizing a more selective criteria for the inherently greater variability of motor unit discharge and EMG signals that has been reported in individuals post‐stroke (Thomas et al., 2002). PSTH of each limb were constructed in a 3 s window around the peaks of the smoothed cumulative spike train of each limb and baseline corrected. Similarly, rectified bipolar EMG signals were low‐pass filtered (second order, 2 Hz cutoff frequency, Butterworth) and the same approach was used to identify EMG amplitude peaks and generate baseline corrected averaged waveform over a 3 s window around the peaks. As previously performed, PSTH and EMG amplitude was estimated as the area under the curve in a window of −200 ms to +200 ms relative to the event. The difference between contralateral and ipsilateral activation amplitude (Amplitude_contra_ – Amplitude_ipsi_) was then computed, with a value of zero representing amplitude of contralateral‐triggered activation equal to ipsilateral‐triggered activation. A value less than zero represents a reduced amplitude of contralateral‐triggered activation compared to ipsilateral triggered activation.

#### Bilateral cross correlation

To further assess bilateral co‐ordination of motor unit and EMG activations, cross correlations were computed on PSTH and EMG waveform averages time‐locked to anterior COP peaks (0°). All signals were baseline corrected and smoothed with a Gaussian kernel of 50 ms. The maximum absolute correlation coefficient and the absolute lag at the time of the maximum absolute correlation coefficient were computed for each trial with no maximum lag enforced. To determine whether there was a significant bilateral co‐ordination between limbs, the cross‐correlation coefficient was compared to a bootstrapped significance threshold that was computed by shuffling the discharge times and EMG time‐series and calculating the cross‐correlation coefficient. The 95th percentile of the bootstrapped cross correlation coefficients over 100 iterations was set as the significance threshold.

### Statistical analysis

For all analyses but the directional‐tuning analysis, we used linear mixed effects models in SPSS, version 26.0.0.1 (IBM Corp., Armonk, NY, USA). Normality of the data were assessed using the Shapiro–Wilk test, as well as by visually examining skewness, kurtosis and histograms. For non‐normal distributions, square root transformations were performed (Kim, 2013; West et al., 1995). If the transformed data remained non‐normal, a generalized linear mixed effects model was used. We compared the BBS of individuals post‐stroke only across visits using a linear mixed effects model with Visit (1, 2) as a fixed effect and Participant as a random effect. We compared the RMS COP displacement (independently for different directions), COP speed (independently for different directions), and cross correlation coefficient and lag using a linear mixed effects model with Group (control, patient), Visit and Task (eyes open, eyes closed) as fixed effects and Participant and Assist level (scored 0–5) as random effect to account for the degree of independence in the tasks. We compared the amplitude of PSTH and EMG independently for each direction, as was performed with the COP metrics, with Limb (control right, control left, paretic, non‐paretic), Visit and Task as fixed effects and Participant and Assist level as random effects. The random structure of all models included random slopes and intercepts to also account for between participant variability in the fixed effects. Ultimately, the model associated with the lowest Bayesian information criterion was retained for the analysis.

Circular statistical analyses were performed on the circular mean angle of PSTH and EMG using the circular statistics MATLAB toolbox (MathWorks Inc., Natick, MA, USA) (Berens, 2009). The Harrison–Kanji test, which is the equivalent of a two‐way analysis of variance (ANOVA) for circular data, was used to identify significant effects of Limb, Visit and Task. All combinations of two‐way interaction were evaluated separately because no three‐way circular ANOVA was available. Where significant interactions were identified, the Watson Williams test (i.e. the equivalent of a one‐way ANOVA for circular data) was used to investigate differences within each factor with significance level of 0.05 with a Bonferroni adjustment (i.e., α/*k*, where *k* is the number of contrasts).

We compared the difference in amplitude of contralateral‐ and ipsilateral‐triggered activation with a linear mixed effects model with Limb (control left/right, control right/left, non‐paretic/paretic, paretic/non‐paretic), Visit and Task as fixed effects and Participant and Assist level as random effects. The selection of the random structure of the models followed the same procedure as above. Significance level was set at 0.05. Bonferroni corrections were used in *post hoc* tests for pairwise comparisons and Cohen's *d* effect sizes were reported with 95% confidence intervals (CI). Outliers exceeding three standard deviations from the mean were excluded.

To determine the relationship between neuromuscular outcomes and functional balance in a stroke population, a linear regression and a repeated measures correlation were performed to examine how the change in BBS was related to the change in any outcome measure that demonstrated a significant change across visits. For this analysis, only individuals with valid data at both visits were included.

## Results

### Balance and centre of pressure

Individuals post‐stroke improved their BBS score from visit 1 (28.43 ± 12.19) to visit 2 (38.57 ± 8.71, *P < *0.001, *d = *0.957, 95% CI = 0.175–1.739) (Fig. 2*A*). Twelve out of 14 participants improved greater than the minimal clinically important difference of 5 points (Tamura et al., 2022). The score of assistance provided by the therapist during the standing task was on average 1.5 ± 1.3 (range 0–5) during the first session and 1.1 ± 1.2 (range 0–4) during the second session. Three and two patients required assistance (score 3–5) during eyes closed and eyes open in week 1, respectively. Two patients required assistance (score 3 to 5) during eyes closed and eyes open in week 2. Six patients required less assistance during the second session while standing eyes closed (−0.5 ± 0.7). Six patients required less assistance during the second session when standing eyes open (–0.4 ± 0.4) and one patient required more assistance during the second session, with a score moving from 1 to 2.

**Figure 2:**
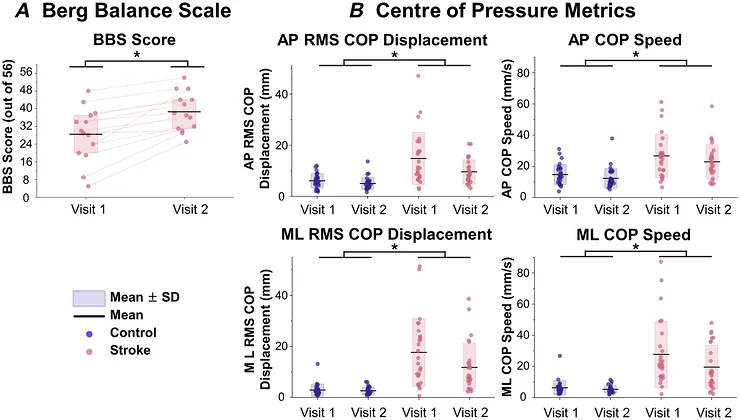
Balance and centre of pressure *A*, Berg Balance Scale (BBS) score out of 56. *B*, anteroposterior (AP) and mediolateral (ML) root mean square (RMS) COP displacement and COP speed are shown. *P < 0.05.

RMS COP displacement in the anteroposterior direction was greater in the stroke group (13.48 ± 6.44 mm) compared to controls (5.53 ± 2.60mm, *P < *0.001, *d = *0.960, 95% CI = 0.565–1.355) and lower at visit 2 (7.14 ± 4.27 mm) compared to visit 1 (10.27±8.53 mm, *P = *0.003, *d = *0.414, 95% CI = 0.004–0.793). Similarly, average COP speed in this direction was greater in the stroke group (24.81 ± 12.57 mm s^−1^) than controls (13.48 ± 6.44 mm s^−1^, *P < *0.001, *d = *0.771, 95% CI = 0.383–1.159) and was lower at visit 2 (17.23 ± 10.21 mm s^−1^) compared to visit 1 (20.50 ± 12.21 mm s^−1^, *P = *0.009, *d = *0.304, 95% CI = −0.072 to 0.680).

RMS COP displacement in the mediolateral direction was greater in the stroke group (14.68 ± 11.77 mm) than controls (2.74 ± 1.92 mm, *P < *0.001, *d = *1.365, 95% CI = 0.949–1.780) and greater with eyes open (9.37 ± 10.79 mm) than eyes closed (7.40 ± 9.43 mm, *P = *0.009, *d = *1.693, 95% CI = 1.257–2.128). Average COP speed in the mediolateral direction was greater in the stroke group (23.64 ± 18.33 mm s^−1^) than controls (5.77 ± 3.57 mm s^−1^, *P < *0.001, *d = *1.242, 95% CI = 0.833–1.650), with greater speeds occurring at visit 1 (V1 = 16.61 ± 18.46 mm s^−1^, V2 = 11.90 ± 12.01 mm s^−1^, *P = *0.004, *d = *0.334, 95% CI = −0.043 to 0.710). Overall, individuals post‐stroke had greater COP displacement and speed than controls (Fig. 2*B*).

### High‐density EMG decomposition

Motor unit yields are shown in Table 2. We excluded one control participant and three stroke participants from the motor unit analyses because no motor units were identified for any muscle.

**Table 2:** Motor unit yield across limbs, groups and visits

		Stroke		Control	
		Paretic	Non‐paretic	Right	Left
**Eyes closed**	**Visit 1**	8.8 ± 10.2	5.0 ± 6.4	11.1 ± 10.7	9.1 ± 9.1
	**Visit 2**	6.1 ± 6.9	8.7 ± 8.0	11.9 ± 8.8	12.8 ± 8.4
**Eyes open**	**Visit 1**	9.2 ± 8.2	5.1 ± 5.6	11.0 ± 11.2	8.3 ± 8.1
	**Visit 2**	6.4 ± 7.0	7.0 ± 7.2	13.6 ± 9.2	12.3 ± 7.4

### Activation amplitude during COP peaks

The amplitude of PSTH and EMG during COP peaks was computed in the anterior, posterior, medial and lateral directions (Fig. 3). For PSTH in the lateral direction, there was an effect of limb (*P = *0.016), with the amplitude in the paretic limb (0.063 ± 0.091 pulses per peak) greater than in the left limb of controls (0.012 ± 0.055 pulses per peak, *P = *0.016, *d = *0.786, 95% CI = 0.309–1.263). No other differences were observed between limbs (all *P* > 0.287). There were neither effects nor interactions in other directions (*P* > 0.111).

**Figure 3:**
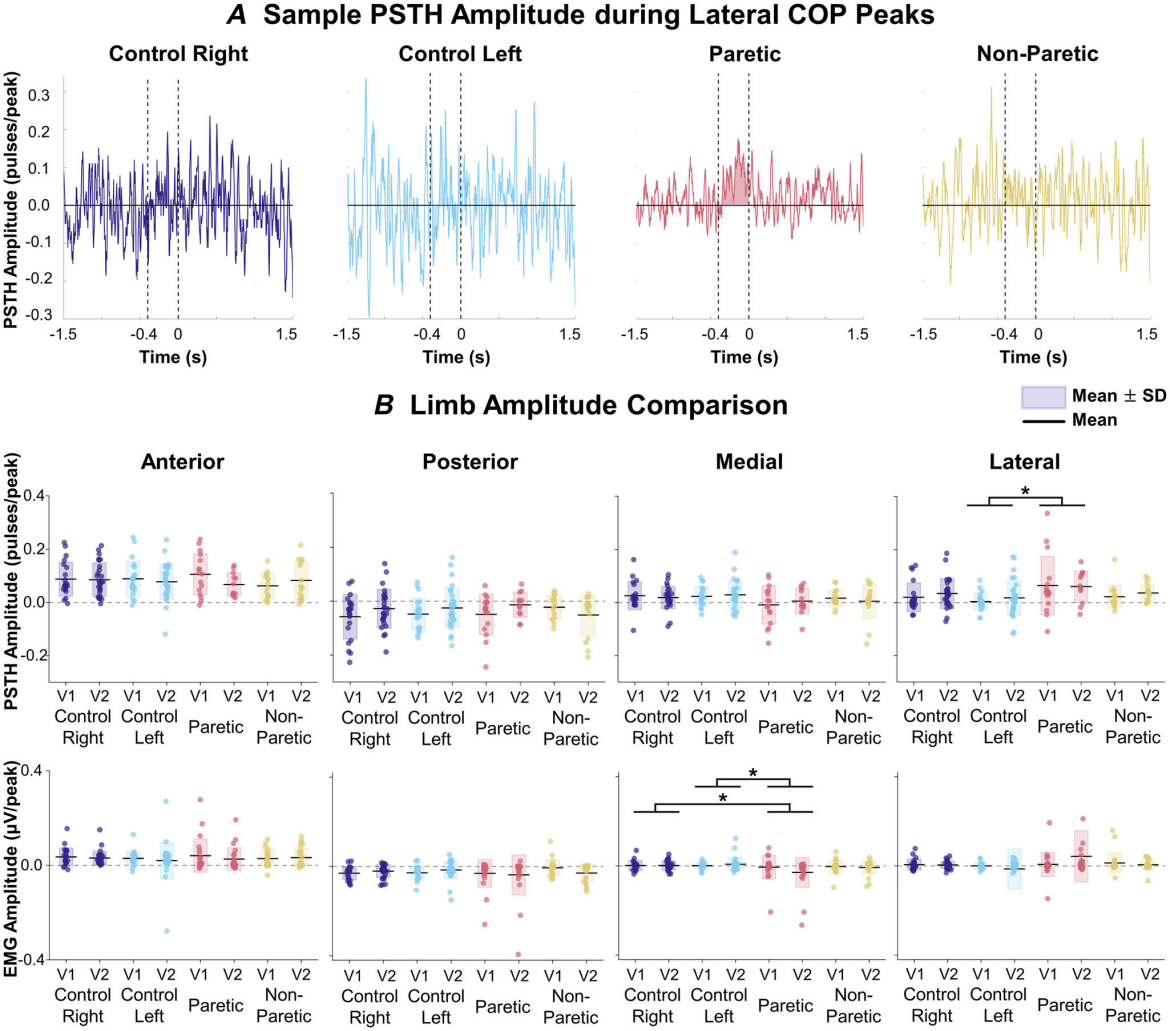
Activation amplitudes in anteroposterior and mediolateral directions *A*, sample peristimulus time histogram (PSTH) amplitude from one control and one stroke participant during lateral centre of pressure (COP) peaks in the eyes closed task during visit 2. Area under the curve between –0.4 s and 0 s relative to the peak in COP. *B*, comparison of PSTH and waveform‐averaged EMG during COP peaks in anterior, posterior, medial and lateral directions. *P < 0.05. V = visit.

For EMG in the medial direction, there was an effect of limb (*P = *0.018), with the amplitude in the paretic limb (–0.018 ± 0.061 µV per peak) lower than in the right limb of controls (0.002 ± 0.020 µV per peak, *P = *0.042, *d = *0.557, 95% CI = 0.160–0.953) and left limb of controls (0.005 ± 0.024 µV per peak, *P = *0.026, *d = *0.590, 95% CI = 0.185–0.996), and with a moderate difference with the non‐paretic limb, albeit non‐significant with our sample size (−0.005 ± 0.029 µV per peak, *P = *0.113, *d = *0.448, 95% CI =  0.037–0.860). There were neither effects nor interactions in other direction (*P>*0.387). Collectively, these results indicate that the activity of the SOL in the paretic limb is modulated with COP displacements along the mediolateral axis.

### Directional tuning

To examine the directional tuning of the activity of the SOL, tuning curves were constructed from motor unit discharge activity (PSTH) and EMG amplitudes time‐locked to COP peaks spanning the transverse plane (Fig. 4). There was an effect of limb on the circular mean angle of these tuning curves derived from both PSTHs (*P = *0.006) and waveform‐averaged EMG amplitudes (*P = *0.029). There were no effects of visit, task or interactions (all *P > *0.201). A Bonferroni correction was used to set the significance threshold at 0.008 (i.e. 0.05/6) for *post hoc* comparisons with Watson‐Williams tests.

**Figure 4:**
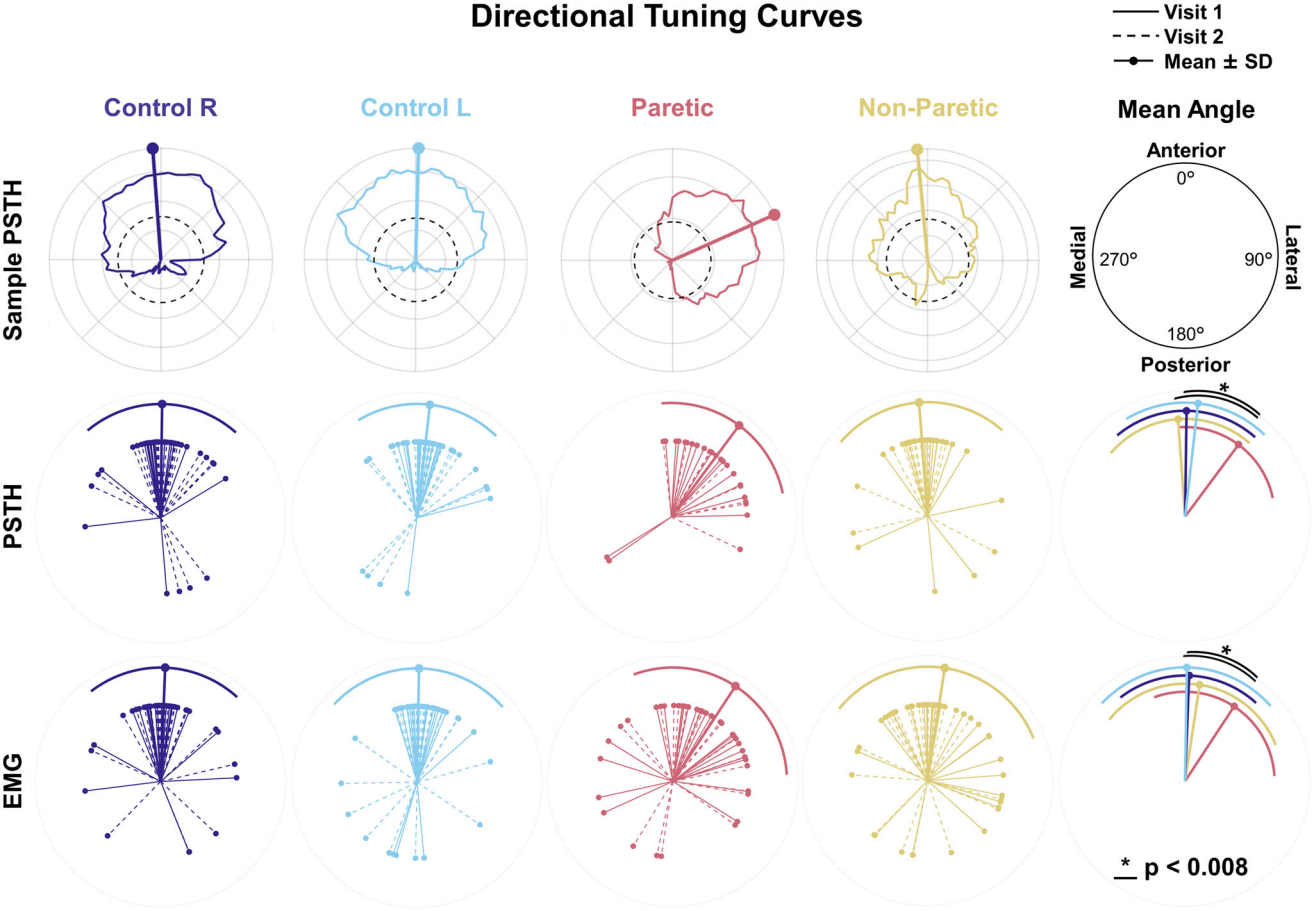
Directional tuning of soleus activity The first row displays directional tuning curves of motor unit activity extracted from peristimulus time histogram (PSTH) from a control and a participant post‐stroke. Additionally, the mean circular angle of the tuning curves extracted from PSTH and EMG plots are shown for each participant for each visit pooled across tasks, and the mean per limb is shown. To compare rotational responses across limbs, rotations were reflected such that 90° always represents lateral tuning and 270° always represents medial tuning. *P < 0.008.

In neurologically intact individuals, the activity of SOL in the control limbs was tuned anteriorly using both PSTH and EMG amplitude, in accordance with the previous work of Zaback & Thompson (2026). The same result was observed in the non‐paretic limbs of individuals post‐stroke. The activity of the SOL in the paretic limb, however, was tuned laterally and anteriorly (Fig. 4). More specifically, for PSTH, the mean angle of the tuning curve was more lateral for the paretic limb (36.56 ± 41.48°) than for the non‐paretic limb (−4.17 ± 45.39°, *P = *0.003, *d = *0.934, 95% CI = 0.400–1.468) and the right control limb (0.79 ± 40.62°, *P = *0.004, *d = *0.874, 95% CI = 0.408–1.340). Despite a moderate effect size, there was no statistically significant difference with the left control limb (6.54 ± 37.56°, *P* = 0.012, *d* = 0.770, 95% CI = 0.294–1.247). For EMG amplitudes, the mean angle of the tuning curve was significantly more lateral for the paretic limb (33.24 ± 53.41°) than for the right control limb (2.27 ± 39.90°, *P* = 0.005, d = 0.662, 95% CI = 0.267–1.056) and the left control limb (0.93 ± 47.75°, *P* = 0.008, *d* = 0.640, 95% CI = 0.235–1.044). There was a moderate difference between the paretic and the non‐paretic limb, albeit non‐significant with our sample size (8.67 ± 59.53°, *P = *0.093, *d = *0.434, 95% CI = 0.025–0.844). Collectively, these results indicate that the activity of the SOL in the paretic limb was tuned laterally and anteriorly, whereas the activity of non‐paretic and control limbs were exclusively tuned anteriorly. This suggests that the greatest activation of the paretic limb occurred during lateral shifts toward the paretic limb.

### Contralateral‐triggered activation

To evaluate the synchronization between limbs, we averaged PSTH and EMG amplitudes for each soleus around phasic bursts from the contralateral soleus (Fig. 5*A* and *B*). We then calculated the difference between contralateral‐ and ipsilateral‐triggered activation amplitudes: zero indicates equal activation, and negative values indicate reduced activation of the limb when peaks of activity are observed in the contralateral limb (Fig. 5*C*).

**Figure 5:**
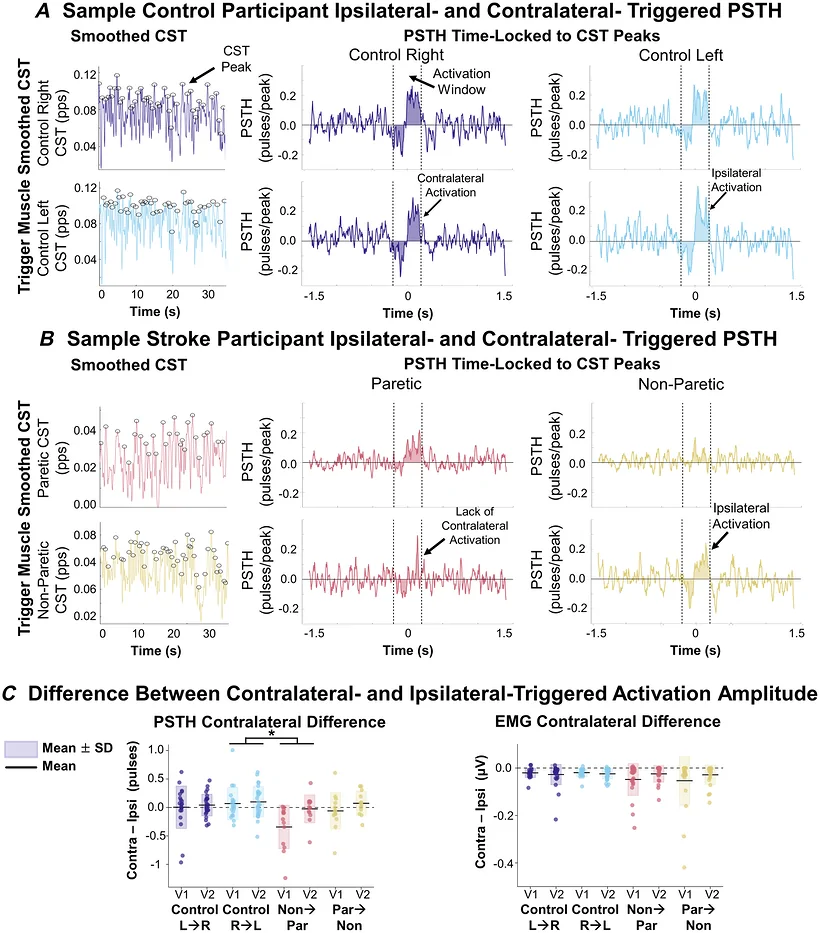
Contralateral‐triggered activation *A*, control right (purple) and left (blue) and (*B*) post‐stroke participant paretic (red) and non‐paretic (yellow) ipsilateral‐ and contralateral‐triggered peristimulus time histogram (PSTH) waveforms during standing with eyes closed (visit 2 for the control and visit 1 for the stroke participant). The smoothed cumulative spike train (CST) is displayed with the peaks shown with circles. The colour code is based on the limb from which the peaks were extracted. As a guide, for the top row of (*A*), the left column is the CST of the control right limb from which the peaks (scatters) were detected. The middle column is the ipsilateral activation of the right limb during right limb peaks in CST. The right column is the contralateral activation of the left limb during right limb peaks in CST. *C*, difference between the amplitude of the contralateral (contra) and ipsilateral (ipsi) triggered activation amplitudes. *P < 0.05. R = right; L = left; Par = paretic; Non = non‐paretic; V = visit; pps = pulses per second.

For PSTH, activations to contralateral peaks were different between visits (*P = *0.011) and limbs (*P = *0.004), without interactions between visits and limbs (*P > *0.199). Specifically, the difference in paretic limb activation during non‐paretic limb peaks (−0.19 ± 0.36 pulses) was lower than the difference observed in the left control limb during right limb peaks (0.08 ± 0.27 pulses, *P = *0.002, *d = *0.734, 95% CI = 0.234–1.234). The differences were not significant with the non‐paretic activation during paretic peaks (0.00 ± 0.28 pulses, *P = *0.082, *d = *0.375, 95% CI = −0.184 to 0.934), and with right control limb activation during left limb peaks (0.03 ± 0.28 pulses, *P = *0.061, *d = *0.585, 95% CI = 0.091–1.080). Thus, activation amplitude of the paretic limb during phasic bursts of the non‐paretic limb was substantially reduced, indicating that the paretic limb failed to co‐ordinate reliably with the non‐paretic limb. For EMG, there was no effect of visit, task, limb or interaction on the difference in amplitudes (*P > *0.245).

### Cross correlation of motor unit and EMG waveform averages

Cross correlation coefficients between SOL activities of each limb and the corresponding absolute lags were computed from PSTH (Fig. 6*A*) and EMG signals. All coefficients were deemed significant compared to the 95^th^ percentile of bootstrapped randomly shuffled signals.

**Figure 6:**
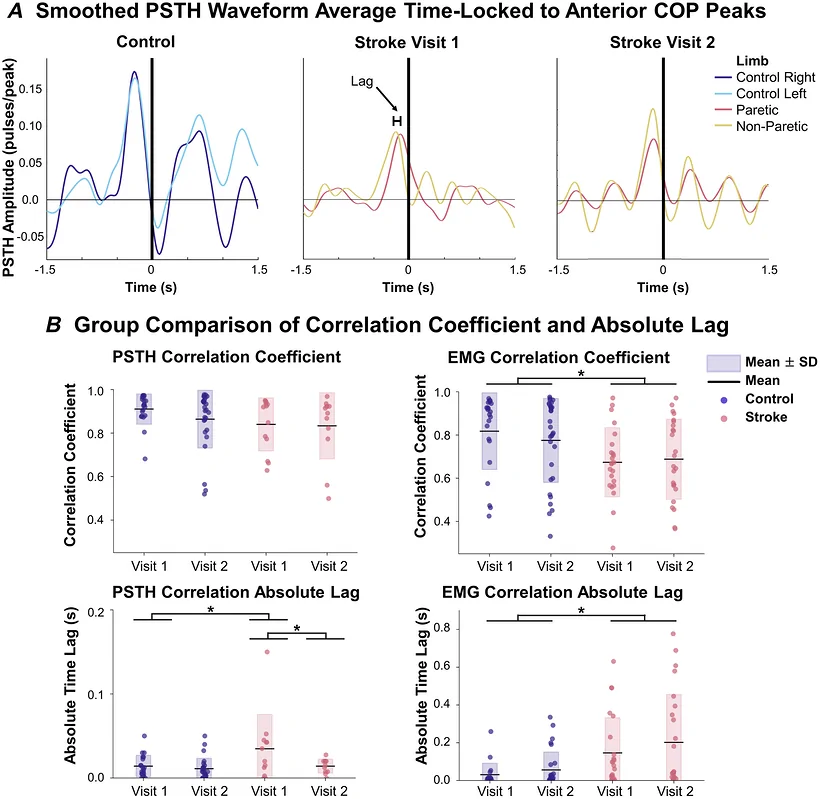
Lag between bilateral soleus activities *A*, bilateral smoothed peristimulus time histogram (PSTH) time‐locked to anterior centre of pressure (COP) peaks for a sample control and stroke participant from visit 1 and visit 2 during the eyes closed task. The graphical display of time lag is depicted, and the black line indicates the time of COP peak. *B*, comparison of the maximum correlation coefficient and absolute time lag of smoothed PSTH and EMG between limbs. *P < 0.05.

For PSTH, there were no effects of group, visit, task or interactions on maximum absolute coefficient (*P > *0.763). For absolute lag, a group × visit interaction (*P = *0.037) (Fig. 6*B*) revealed that absolute lag was greater for the stroke group at visit 1 (0.03 ± 0.04 s) compared to visit 2 (0.01 ± 0.01 s, *P = *0.009, *d = *0.912, 95% CI = −0.009 to 1.833) and was greater for the stroke group at visit 1 compared to controls (0.01 ± 0.01 s, *P = *0.008, *d = *4.454, 95% CI = 3.054–5.853). However, absolute lag of the stroke group was no longer different from controls at visit 2 (0.01 ± 0.01 s, *P = *0.491, *d = *0.319, 95% CI = −0.438 to 1.077), whereas absolute lag did not differ across visits for controls (*P = *0.912, *d = *0.021, 95% CI = −0.608 to 0.651).

For EMG, there was an effect of group (*P = *0.005) (Fig. 6*B*), but no effects of visit, task or interactions on maximum absolute coefficient (*P>*0.170). Controls demonstrated greater correlation coefficients (0.79 ± 0.19) compared to the stroke group (0.68 ± 0.17, *P = *0.005, *d = *0.381, 95% CI = −0.017 to 0.779). The stroke group demonstrated higher absolute lag (0.17±0.22s) than the control group (0.05 ± 0.08 s, *P = *0.001, *d = *0.916, 95% CI = 0.461–1.371) (Fig. 6*B*).

Collectively, these results indicate greater lag, and therefore reduced co‐ordination, between limbs after a stroke. Importantly, when assessing lags on activity in PSTH, the stroke group exhibited an improvement such that the bilateral synchronization improved from visit 1 to visit 2 to comparable levels to controls.

### Regressions with changes in clinical outcomes

Only PSTH absolute lag demonstrated a significant longitudinal change for stroke participants. Therefore, the relationship between the change in PSTH absolute lag and the change in functional balance score (i.e. BBS score) for stroke participants was assessed via linear regressions. Data were averaged across tasks per participant as task effect was absent. A linear regression between the changes in lag between visits and the change in BBS score between visits revealed a significant negative correlation (*R = −*0.94, *P *< 0.001). This was further confirmed by a repeated measures correlation analysis with a significant negative correlation between both variables (*R = −*0.78; *P = *0.002). Thus, an increase in BBS score was accompanied by a decrease in lag (or an increase in synchronization).

## Discussion

In the present study, we quantified the bilateral co‐ordination of soleus motor units during standing balance tasks in individuals in the early subacute phase post‐stroke. Although clinical assessments of balance and COP displacements improved between visits, individuals post‐stroke continued to exhibit greater COP variability than controls. Furthermore, individuals post‐stroke demonstrated differences in directional tuning and synchronization of their soleus activity. Thus, although the soleus of the non‐paretic limb was activated along the anteroposterior axis, similar to controls, the soleus of the paretic limb exhibited preferential activation along the mediolateral axis. The bilateral synchronization of soleus improved with training, and this improvement was shown to be related to improvement in functional balance, emphasizing its importance to effective balance recovery.

### Altered directional tuning of activity of the paretic limb

The soleus plays an essential role in balance. Its tonic activity during weight‐bearing is critical for maintaining upright stance by counteracting the gravitational torque acting on the centre of mass (Di Giulio et al., 2009; Loram et al., 2004). Previous work that has examined the directional tuning of the soleus, either through analysis of activation amplitude in response to multi‐directional perturbations or through natural COP oscillations during unperturbed standing, has shown that the soleus activity is primarily modulated with movements of the COP or centre of mass along the sagittal plane (Loram et al., 2005; Ting, 2007; Winter et al., 1993; Winter et al., 2003; Zaback & Thompson, 2026). Cohen et al. (2021; 2023a) further demonstrated in neurologically intact individuals that some motor units in the soleus and gastrocnemius medialis respond broadly across directions, whereas others show direction‐specific tuning. The results of our study corroborated these previous findings, with motor unit and EMG activity directionally tuned to anterior COP peaks in neurologically intact individuals. Anteroposterior sway is indeed controlled through modulation of ankle plantarflexor and dorsiflexor torques, whereas mediolateral sway is generally controlled by loading and unloading the hip rather than by inverting and everting the ankle joints (Day et al., 1993; Winter et al., 1993; Winter et al., 1996). It is noteworthy that the mean angles of the tuning curve observed here exhibited higher variability than those measured by Zaback & Thompson (2026) (SD: 37–47° vs. 17.7°). This difference may result from differences in demographics for the control group (eight males/females, mean age: 61.2 ± 14.7 years here vs. 10 males; mean age: 29.9 ± 7.2 years in Zaback & Thompson, 2026) because older individuals often experience impairments in balance control (Granacher et al., 2011; Matsuda et al., 2025). Another factor explaining this variability could be the duration of trials used to construct the tuning curves, which was limited here to 30 s because of the inability of our patients to maintain postures for long periods of time. However, the mean angles of control and non‐paretic limbs align with the previous study performed in neurologically intact individuals (Zaback & Thompson, 2026), suggesting the robustness of the method with shorter trials.

In patients, although the non‐paretic limb preserved the directional control of soleus with anteriorly tuned activation, the paretic limb displayed an abnormal directional tuning, with modulations of the soleus activation along the mediolateral axis (Fig. 3) and greater activation during anterior and lateral COP movement (∼40° from anterior) (Fig. 4). This abnormal modulation along the mediolateral axis may be the result of a couple of mechanisms. First, previous work has shown that the torque necessary to recover from perturbations in patients post‐stroke is generated more slowly when directed toward the paretic limb (Palmer et al., 2025). This weakened response likely results from compressed rate coding, impaired recruitment and fatigue (Beauchamp et al., 2023; Chou et al., 2013; Hu et al., 2015; Hu et al., 2016; Kallenberg & Hermens, 2011; Li et al., 2015; McNulty et al., 2014; Miller, Thompson et al., 2014; Mottram et al., 2014; Negro et al., 2020; Tacca et al., 2026). Therefore, in such a situation, the nervous system may activate the paretic limb only for the most destabilizing perturbations for which the non‐paretic limb cannot fully compensate, such as lateral shifts. Second, individuals post‐stroke exhibit impaired sensorimotor processing and spinal reflexes (Aman et al., 2014; Chia et al., 2019; Gorst et al., 2019; Johnson et al., 2025; Wutzke et al., 2013), which are critical for postural control. This may result in abnormal directional tuning of the paretic limb.

It is noteworthy that the physical therapist assistance may have also impacted these outcomes because even short touch assistance from the therapist likely reduced COP displacement and speed around COP peaks in some directions. However, the touch assistance was only performed to prevent a fall of the patient, and the three patients with score of 3–5 still exhibited higher COP displacement than patients with a score of 0–2 (e.g. during week 1: 29.9 ± 6.0 mm and 30.5 ± 12.1 mm in AP and ML directions vs. 13.3 ± 10.6 mm and 14.6 ± 11.8 mm, respectively). Moreover, the level of assistance did not change over time (average change: −0.43 ± 0.63 points out of 5, *P* = 0.251) and both groups of patients reduced their COP displacement between visits.

### Reduced bilateral synchronization post‐stroke

The bilateral co‐ordination of soleus muscles was also disrupted temporally, with less synchronization of their activation. Anterior sway generally induces greater common drive between soleus muscles compared to backward sway (Mochizuki et al., 2007), whereas greater instability is associated with increased lag and reduced correlation between bilateral soleus muscles (Promsri, 2022). In the present study, the cross‐correlation analysis also revealed greater lag between limbs during anterior COP peaks in individuals post‐stroke compared to controls. This result was confirmed by also evaluating EMG amplitude of soleus muscles during peaks in activity in the contralateral limb. There was a lower activity in the soleus of the paretic limb during peaks of activity of the other soleus compared to its own peaks of activity. In controls, there were no differences in amplitude when triggered by the activity in the contralateral side or by its own peaks of activity. Note that because of the lack of normalization of the EMG amplitude, we cannot interpret EMG amplitudes as an indicator of muscle activation and cannot compare the raw values between participants and sessions. Here, we only interpret the relative differences in amplitude within the same muscle using the entire grid of electrodes.

Again, the lack of synchronization in patients may result from disrupted processing and transmission of proprioceptive information between limbs. In neurologically intact individuals, the disruption of proprioceptive feedback with a vibratory stimulus decreases the common drive between bilateral soleus muscles (Mochizuki et al., 2007). In patients post‐stroke, reflex triggering via non‐noxious electrical nerve stimulation is also disrupted when the triggering is performed on the paretic limb (Dietz & Schrafl‐Altermatt, 2016). In the context of our study, the altered processing of sensory information for bilateral motor co‐ordination during standing may impair the ability of patients to generate synchronous stabilizing torques with their soleus.

Bulbospinal pathways, such as vestibulospinal and reticulospinal tracts, are important for modulation of soleus motor unit activity in neurologically intact individuals (Monsour et al., 2012), making their function critical for balance. A stroke may alter the bilateral gains and descending commands of these pathways and have debilitating effects on motor control and co‐ordination (Marsden, 2005; Mccall et al., 2017; Miller, Klein et al., 2014). Previous research has shown that the activation of brainstem pathways following stroke led to hyperactive force responses on the non‐paretic limb (Marsden, 2005), suggesting asymmetrical brainstem activation. Overall, it has been suggested that the modification of balance may be mediated by a combination of central and peripheral systems, where central structures may modulate feedback loops based on task demands (Welch & Ting, 2014). Therefore, impairments in descending commands and proprioception after stroke may have contributed to the abnormal balance observed in this study. Future work is necessary to provide a more direct understanding of the neural circuits underlying impaired balance post‐stroke and how they interact at the different levels.

### Motor unit co‐ordination predictor of balance function

In addition to characterizing deficits in neural co‐ordination during the early subacute phase post‐stroke, we aimed to evaluate the relationship between changes in these neural measures and functional recovery, as assessed by licensed physical therapists using the widely used balance assessment, the BBS. A regression between the change in BBS and the change in synchronization of motor unit activity (PSTH absolute lag) between visits was assessed becaued this was the only metric to demonstrate an improvement across visits. This analysis revealed that a change in synchronization was strongly associated with a change in BBS score (*R = −*0.94; *P *< 0.001), suggesting that individuals who improve their bilateral synchronization also achieve improved functional balance.

Thus, PSTH‐derived metrics may be considered as a biomarker when assessing patients’ control of balance. However, the application of such approaches is currently limited to high‐quality recordings capable of extracting multiple motor unit firings in patients. For example, it is noteworthy that our regression analysis only included individuals with motor units extracted in both visits, with three individuals in the stroke group excluded from all motor unit analyses because of the inability to extract any motor units. The absence of extracted motor units may be explained not only by physiological factors, such as low muscle activation or reduced neural drive, but also poor signal quality, being particularly present after stroke. Therefore, to extend the applicability of motor unit‐based clinical analyses, future work must continue to improve high‐density EMG sensors, extend investigations to intramuscular EMG, and enhance decomposition techniques to increase motor unit yields across wider populations. Alternatively, EMG‐derived parameters that do not rely on decomposed signals could also become biomarkers, although our study was probably underpowered to demonstrate this.

The synchronous behaviour of motor units may be facilitated by preserved neural pathways in the higher functioning individuals. Recent studies have investigated altered behaviour of motor unit and how the altered behaviour correlated with functional impairments (e.g. Fugl‐Meyer, fatigue, walking speed) (Hu et al., 2016; Kallenberg & Hermens, 2009; Li et al., 2024; McManus et al., 2017; Negro et al., 2020; Tacca et al., 2026). However, only Li et al. (2024) evaluated longitudinal relationships between motor unit number estimates/F‐waves and upper extremity Fugl‐Meyer score, highlighting a critical next step in elucidating the neural mechanisms underlying the impaired motor control observed in this study. Future work should also combine recording modalities to evaluate changes in motor function at multiple levels (e.g. peripheral, spinal, brainstem, cortical) to more effectively map the altered neural networks responsible for post‐stroke impaired balance.

## Additional information

## Competing interests

The authors declare that they have no competing interests.

## Author contributions

Experiments were performed at Shirley Ryan AbilityLab. J.T.L., A.J., S.A. and J.L.P. contributed to the conception and design of the work. J.T.L., X.S.Y., A.J., R.M., S.A. and J.L.P. contributed to the acquisition of data. J.T.L., X.S.Y., M.Z., C.K.T., D.F., S.A. and J.L.P. contributed to the analysis and interpretation of the data. All authors contributed to drafting and revising the manuscript. Additionally, all authors approved the final version of the manuscript submitted for publication, and agree to be accountable for all aspects of the work in ensuring that questions related to the accuracy or integrity of any part of the work are appropriately investigated and resolved. All persons designated as authors qualify for authorship, and all those who qualify for authorship are listed.

## Funding

This research was funded by Northwestern University and Shirley Ryan AbilityLab. Simon Avrillon is supported by the French National Research Agency through Nantes Excellence Trajectory (NExT, ANR‐16‐IDEX‐0007) and the French National Research Agency (Neuromotor, ANR‐24‐CE17‐5805).

## Generative AI statement

No generative AI tools were used in the preparation of this manuscript.

## Supporting information


Peer Review History

## Data Availability

The data supporting the findings of this study are available from the corresponding author upon reasonable request.

## References

[tjp70847-bib-0001] Aman, J. E. , Elangovan, N. , Yeh, I. L. , & Konczak, J. (2014). The effectiveness of proprioceptive training for improving motor function: A systematic review. Frontiers in Human Neuroscience, 8, 1075. 10.3389/fnhum.2014.01075 PMC4309156 25674059

[tjp70847-bib-0002] Arya, K. N. , & Pandian, S. (2014). Interlimb neural coupling: Implications for poststroke hemiparesis. Annals of Physical and Rehabilitation Medicine, 57(9–10), 696–713. 10.1016/j.rehab.2014.06.003 25262645

[tjp70847-bib-0003] Avrillon, S. , Hug, F. , Baker, S. N. , Gibbs, C. , & Farina, D. (2024). Tutorial on MUedit: An open‐source software for identifying and analysing the discharge timing of motor units from electromyographic signals. Journal of Electromyography and Kinesiology, 77, 102886. 10.1016/j.jelekin.2024.102886 38761514

[tjp70847-bib-0004] Beauchamp, J. A. , Hassan, A. S. , Mcpherson, L. M. , Negro, F. , Pearcey, G. E. P. , Cummings, M. , Heckman, C. , & Dewald, J. P. A. (2023). Motor unit firing rate modulation is more impaired during flexion synergy‐driven contractions of the biceps brachii in chronic stroke. Cold Spring Harbor Laboratory.

[tjp70847-bib-0005] Berens, P. (2009). CircStat: A MATLAB toolbox for circular statistics. Journal of Statistical Software 31(10), 1–21.

[tjp70847-bib-0006] Boonstra, T. W. , Daffertshofer, A. , Roerdink, M. , Flipse, I. , Groenewoud, K. , & Beek, P. J. (2009). Bilateral motor unit synchronization of leg muscles during a simple dynamic balance task. European Journal of Neuroscience, 29(3), 613–622. 10.1111/j.1460-9568.2008.06584.x 19175407

[tjp70847-bib-0007] Brott, T. , Adams, H. P. , Olinger, C. P. , Marler, J. R. , Barsan, W. G. , Biller, J. , Spilker, J. , Holleran, R. , Eberle, R. , & Hertzberg, V. (1989). Measurements of acute cerebral infarction: A clinical examination scale. Stroke; A Journal of Cerebral Circulation, 20(7), 864–870. 10.1161/01.str.20.7.864 2749846

[tjp70847-bib-0008] Campbell, B. C. V. , & Khatri, P. (2020). Stroke. Lancet, 396(10244), 129–142. 10.1016/S0140-6736(20)31179-X 32653056

[tjp70847-bib-0009] Castronovo, A. M. , Negro, F. , Conforto, S. , & Farina, D. (2015). The proportion of common synaptic input to motor neurons increases with an increase in net excitatory input. Journal of Applied Physiology (1985), 119(11), 1337–1346. 10.1152/japplphysiol.00255.2015 26404614

[tjp70847-bib-0010] Chia, F. S. , Kuys, S. , & Low Choy, N. (2019). Sensory retraining of the leg after stroke: Systematic review and meta‐analysis. Clinical Rehabilitation, 33(6), 964–979. 10.1177/0269215519836461 PMC6557007 30897960

[tjp70847-bib-0011] Chou, L.‐W. , Palmer, J. A. , Binder‐Macleod, S. , & Knight, C. A. (2013). Motor unit rate coding is severely impaired during forceful and fast muscular contractions in individuals post stroke. Journal of Neurophysiology, 109(12), 2947–2954. 10.1152/jn.00615.2012 PMC3680820 23554434

[tjp70847-bib-0012] Cohen, J. W. , Vieira, T. , Ivanova, T. D. , Cerone, G. L. , & Garland, S. J. (2021). Maintenance of standing posture during multi‐directional leaning demands the recruitment of task‐specific motor units in the ankle plantarflexors. Experimental Brain Research, 239(8), 2569–2581. 10.1007/s00221-021-06154-0 34191118

[tjp70847-bib-0013] Cohen, J. W. , Vieira, T. M. , Ivanova, T. D. , & Garland, S. J. (2023a). Differential behavior of distinct motoneuron pools that innervate the triceps surae. Journal of Neurophysiology, 129(1), 272–284. 10.1152/jn.00336.2022 36475977

[tjp70847-bib-0014] Cohen, J. W. , Vieira, T. M. , Ivanova, T. D. , & Garland, S. J. (2023b). Regional recruitment and differential behavior of motor units during postural control in older adults. Journal of Neurophysiology, 130(5), 1321–1333. 10.1152/jn.00068.2023 PMC10972635 37877159

[tjp70847-bib-0015] Day, B. L. , Steiger, M. J. , Thompson, P. D. , & Marsden, C. D. (1993). Effect of vision and stance width on human body motion when standing: Implications for afferent control of lateral sway. The Journal of Physiology, 469(1), 479–499. 10.1113/jphysiol.1993.sp019824 PMC1143881 8271209

[tjp70847-bib-0016] Del Vecchio, A. , Holobar, A. , Falla, D. , Felici, F. , Enoka, R. M. , & Farina, D. (2020). Tutorial: Analysis of motor unit discharge characteristics from high‐density surface EMG signals. Journal of Electromyography and Kinesiology, 53, 102426. 10.1016/j.jelekin.2020.102426 32438235

[tjp70847-bib-0017] Di Giulio, I. , Maganaris, C. N. , Baltzopoulos, V. , & Loram, I. D. (2009). The proprioceptive and agonist roles of gastrocnemius, soleus and tibialis anterior muscles in maintaining human upright posture. The Journal of Physiology, 587(10), 2399–2416. 10.1113/jphysiol.2009.168690 PMC2697307 19289550

[tjp70847-bib-0018] Dickstein, R. , & Abulaffio, N. (2000). Postural sway of the affected and nonaffected pelvis and leg in stance of hemiparetic patients. Archives of Physical Medicine and Rehabilitation, 81(3), 364–367. 10.1016/s0003-9993(00)90085-6 10724084

[tjp70847-bib-0019] Dietz, V. , & Schrafl‐Altermatt, M. (2016). Control of functional movements in healthy and post‐stroke subjects: Role of neural interlimb coupling. Clinical neurophysiology : official journal of the International Federation of Clinical Neurophysiology, 127(5), 2286–2293. 10.1016/j.clinph.2016.02.014 27072101

[tjp70847-bib-0020] Garland, S. , Willems, D. A. , Ivanova, T. D. , & Miller, K. J. (2003). Recovery of standing balance and functional mobility after stroke11No commercial party having a direct financial interest in the results of the research supporting this article has or will confer a benefit upon the author(s) or upon any organization with which the author(s) is/are associated. Archives of Physical Medicine and Rehabilitation, 84, 1753–1759. 10.1016/j.apmr.2003.03.002 14669179

[tjp70847-bib-0021] Garland, S. J. , Ivanova, T. D. , & Mochizuki, G. (2007). Recovery of standing balance and health‐related quality of life after mild or moderately severe stroke. Archives of Physical Medicine and Rehabilitation, 88(2), 218–227. 10.1016/j.apmr.2006.11.023 17270520

[tjp70847-bib-0022] Garland, S. J. , Pollock, C. L. , & Ivanova, T. D. (2014). Could motor unit control strategies be partially preserved after stroke? Frontiers in Human Neuroscience, 8, 864. 10.3389/fnhum.2014.00864 PMC4215611 25400568

[tjp70847-bib-0023] Gibbs, J. , Harrison, L. M. , & Stephens, J. A. (1995). Organization of inputs to motoneurone pools in man. The Journal of Physiology, 485(1), 245–256. 10.1113/jphysiol.1995.sp020727 PMC1157987 7658378

[tjp70847-bib-0024] Gorst, T. , Rogers, A. , Morrison, S. C. , Cramp, M. , Paton, J. , Freeman, J. , & Marsden, J. (2019). The prevalence, distribution, and functional importance of lower limb somatosensory impairments in chronic stroke survivors: A cross sectional observational study. Disability and Rehabilitation, 41(20), 2443–2450. 10.1080/09638288.2018.1468932 29726732

[tjp70847-bib-0025] Granacher, U. , Bridenbaugh, S. A. , Muehlbauer, T. , Wehrle, A. , & Kressig, R. W. (2011). Age‐related effects on postural control under multi‐task conditions. Gerontology, 57(3), 247–255. 10.1159/000322196 20980734

[tjp70847-bib-0026] Héroux, M. E. , Dakin, C. J. , Luu, B. L. , Inglis, J. T. , & Blouin, J.‐S. (2014). Absence of lateral gastrocnemius activity and differential motor unit behavior in soleus and medial gastrocnemius during standing balance. Journal of Applied Physiology, 116, 140–148. 10.1152/japplphysiol.00906.2013 PMC3921363 24311748

[tjp70847-bib-0027] Holobar, A. , Minetto, M. A. , & Farina, D. (2014). Accurate identification of motor unit discharge patterns from high‐density surface EMG and validation with a novel signal‐based performance metric. Journal of Neural Engineering, 11(1), 016008. 10.1088/1741-2560/11/1/016008 24654270

[tjp70847-bib-0028] Hu, X. , Suresh, A. K. , Rymer, W. Z. , & Suresh, N. L. (2015). Assessing altered motor unit recruitment patterns in paretic muscles of stroke survivors using surface electromyography. Journal of Neural Engineering, 12(6), 066001. 10.1088/1741-2560/12/6/066001 PMC5439428 26402920

[tjp70847-bib-0029] Hu, X. , Suresh, A. K. , Rymer, W. Z. , & Suresh, N. L. (2016). Altered motor unit discharge patterns in paretic muscles of stroke survivors assessed using surface electromyography. Journal of Neural Engineering, 13(4), 046025. 10.1088/1741-2560/13/4/046025 PMC5439147 27432656

[tjp70847-bib-0030] Hug, F. , Avrillon, S. , Del Vecchio, A. , Casolo, A. , Ibanez, J. , Nuccio, S. , Rossato, J. , Holobar, A. , & Farina, D. (2021). Analysis of motor unit spike trains estimated from high‐density surface electromyography is highly reliable across operators. Journal of Electromyography and Kinesiology, 58, 102548. 10.1016/j.jelekin.2021.102548 33838590

[tjp70847-bib-0031] Hug, F. , Del Vecchio, A. , Avrillon, S. , Farina, D. , & Tucker, K. (2021). Muscles from the same muscle group do not necessarily share common drive: Evidence from the human triceps surae. Journal of Applied Physiology, 130(2), 342–354. 10.1152/japplphysiol.00635.2020 33242301

[tjp70847-bib-0032] Johnson, C. A. , Biswas, P. , Tapia, R. , See, J. , Dodakian, L. , Chan, V. , Wang, P. T. , Nenadic, Z. , Do, A. H. , & Reinkensmeyer, D. J. (2025). The weak relationship between ankle proprioception and gait speed after stroke: A robotic assessment study. Neurorehabilitation and Neural Repair, 39(12), 1031–1045. 10.1177/15459683251369497 PMC12443327 40956975

[tjp70847-bib-0033] Kallenberg, L. A. , & Hermens, H. J. (2009). Motor unit properties of biceps brachii in chronic stroke patients assessed with high‐density surface EMG. Muscle & Nerve, 39(2), 177–185. 10.1002/mus.21090 19034958

[tjp70847-bib-0034] Kallenberg, L. A. C. , & Hermens, H. J. (2011). Motor unit properties of biceps brachii during dynamic contractions in chronic stroke patients. Muscle & Nerve, 43(1), 112–119. 10.1002/mus.21803 20928907

[tjp70847-bib-0035] Karbasforoushan, H. , Cohen‐Adad, J. , & Dewald, J. P. A. (2019). Brainstem and spinal cord MRI identifies altered sensorimotor pathways post‐stroke. Nature Communications 10(1), 3524. 10.1038/s41467-019-11244-3 PMC6684621 31388003

[tjp70847-bib-0036] Khan, F. , & Chevidikunnan, M. F. (2021). Prevalence of balance impairment and factors associated with balance among patients with stroke. A cross sectional retrospective case control study. Healthcare, 9(3), 320. 10.3390/healthcare9030320 PMC7998930 33805643

[tjp70847-bib-0037] Kim, H.‐Y. (2013). Statistical notes for clinical researchers: Assessing normal distribution (2) using skewness and kurtosis. Restorative dentistry & endodontics, 38(1), 52. 10.5395/rde.2013.38.1.52 PMC3591587 23495371

[tjp70847-bib-0038] Li, X. , Holobar, A. , Gazzoni, M. , Merletti, R. , Rymer, W. Z. , & Zhou, P. (2015). Examination of poststroke alteration in motor unit firing behavior using high‐density surface EMG decomposition. Institute of Electrical and Electronics Engineers Transactions on Bio‐Medical Engineering, 62(5), 1242–1252. 10.1109/TBME.2014.2368514 PMC4406795 25389239

[tjp70847-bib-0039] Li, X. , Shao, Z. , Li, Z. , Wei, X. , Zong, L. , Wang, P. , Zhou, T. , & Wang, H. (2024). The relationship between the functional status of upper extremity motor neurons and motor function and prognosis in stroke patients. Frontiers in Neurology 15, 1466252. 10.3389/fneur.2024.1466252 PMC11451047 39372704

[tjp70847-bib-0040] Li, X. , Wang, Y.‐C. , Suresh, N. L. , Rymer, W. Z. , & Zhou, P. (2011). Motor unit number reductions in paretic muscles of stroke survivors. Institute of Electrical and Electronics Engineers Transactions on Information Technology in Biomedicine, 15, 505–512. 10.1109/TITB.2011.2140379 21478079

[tjp70847-bib-0041] Loram, I. D. , Maganaris, C. N. , & Lakie, M. (2004). Paradoxical muscle movement in human standing. The Journal of Physiology, 556(3), 683–689. 10.1113/jphysiol.2004.062398 PMC1664994 15047776

[tjp70847-bib-0042] Loram, I. D. , Maganaris, C. N. , & Lakie, M. (2005). Human postural sway results from frequent, ballistic bias impulses by soleus and gastrocnemius. The Journal of Physiology, 564(1), 295–311. 10.1113/jphysiol.2004.076307 PMC1456055 15661824

[tjp70847-bib-0043] Lukács, M. (2005). Electrophysiological signs of changes in motor units after ischaemic stroke. Clinical Neurophysiology : Official Journal of the International Federation of Clinical Neurophysiology, 116(7), 1566–1570. 10.1016/j.clinph.2005.04.005 15905127

[tjp70847-bib-0044] Marsden, J. F. (2005). The vestibular control of balance after stroke. Journal of Neurology, Neurosurgery & Psychiatry, 76(5), 670–679. 10.1136/jnnp.2004.046565 PMC1739624 15834025

[tjp70847-bib-0045] Matsuda, K. , Murai, I. , Okoba, R. , Ikeda, T. , & Takano, Y. (2025). Differences in postural control associated with aging and executive function. Cureus, 17, e81771. 10.7759/cureus.81771 PMC12054940 40330354

[tjp70847-bib-0046] Mccall, A. A. , Miller, D. M. , & Yates, B. J. (2017). Descending influences on vestibulospinal and vestibulosympathetic reflexes. Frontiers in Neurology, 8, 112. 10.3389/fneur.2017.00112 PMC5366978 28396651

[tjp70847-bib-0047] McManus, L. , Hu, X. , Rymer, W. Z. , Suresh, N. L. , & Lowery, M. M. (2017). Motor unit activity during fatiguing isometric muscle contraction in hemispheric stroke survivors. Frontiers in Human Neuroscience, 11, 569. 10.3389/fnhum.2017.00569 PMC5705653 29225574

[tjp70847-bib-0048] McNulty, P. A. , Lin, G. , & Doust, C. G. (2014). Single motor unit firing rate after stroke is higher on the less‐affected side during stable low‐level voluntary contractions. Frontiers in Human Neuroscience, 8, 518. 10.3389/fnhum.2014.00518 PMC4102083 25100969

[tjp70847-bib-0049] Miller, D. M. , Klein, C. S. , Suresh, N. L. , & Rymer, W. Z. (2014). Asymmetries in vestibular evoked myogenic potentials in chronic stroke survivors with spastic hypertonia: Evidence for a vestibulospinal role. Clinical Neurophysiology, 125(10), 2070–2078. 10.1016/j.clinph.2014.01.035 PMC4162855 24680197

[tjp70847-bib-0050] Miller, L. C. , Thompson, C. K. , Negro, F. , Heckman, C. J. , Farina, D. , & Dewald, J. P. (2014). High‐density surface EMG decomposition allows for recording of motor unit discharge from proximal and distal flexion synergy muscles simultaneously in individuals with stroke. Annual International Conference of the IEEE Engineering in Medicine and Biology Society. Annual International Conference, 2014, 5340–5344. 10.1109/EMBC.2014.6944832 PMC4410846 25571200

[tjp70847-bib-0051] Mizrahi, J. , Solzi, P. , Ring, H. , & Nisell, R. (1989). Postural stability in stroke patients: Vectorial expression of asymmetry, sway activity and relative sequence of reactive forces. Medical & Biological Engineering & Computing, 27(2), 181–190. 10.1007/BF02446228 2601436

[tjp70847-bib-0052] Mochizuki, G. , Ivanova, T. D. , & Garland, S. J. (2007). Factors affecting the common modulation of bilateral motor unit discharge in human soleus muscles. Journal of Neurophysiology, 97(6), 3917–3925. 10.1152/jn.01025.2006 17409169

[tjp70847-bib-0053] Monsour, M. , Ivanova, T. D. , Wilson, T. D. , & Garland, S. J. (2012). Influence of vestibular afferent input on common modulation of Human soleus motor units during standing. Motor Control, 16(4), 466–479. 10.1123/mcj.16.4.466 23162063

[tjp70847-bib-0054] Mottram, C. J. , Heckman, C. J. , Powers, R. K. , Rymer, W. Z. , & Suresh, N. L. (2014). Disturbances of motor unit rate modulation are prevalent in muscles of spastic‐paretic stroke survivors. Journal of Neurophysiology, 111(10), 2017–2028. 10.1152/jn.00389.2013 PMC4044339 24572092

[tjp70847-bib-0055] Murphy, S. A. , Negro, F. , Farina, D. , Onushko, T. , Durand, M. , Hunter, S. K. , Schmit, B. D. , & Hyngstrom, A. (2018). Stroke increases ischemia‐related decreases in motor unit discharge rates. Journal of Neurophysiology, 120(6), 3246–3256. 10.1152/jn.00923.2017 PMC6337044 30379629

[tjp70847-bib-0056] Negro, F. , Bathon, K. E. , Nguyen, J. N. , Bannon, C. G. , Orizio, C. , Hunter, S. K. , & Hyngstrom, A. S. (2020). Impaired firing behavior of individually tracked paretic motor units during fatiguing contractions of the dorsiflexors and functional implications post stroke. Frontiers in Neurology, 11, 540893. 10.3389/fneur.2020.540893 PMC7658471 33192970

[tjp70847-bib-0057] Negro, F. , Muceli, S. , Castronovo, A. M. , Holobar, A. , & Farina, D. (2016). Multi‐channel intramuscular and surface EMG decomposition by convolutive blind source separation. Journal of Neural Engineering, 13(2), 026027. 10.1088/1741-2560/13/2/026027 26924829

[tjp70847-bib-0058] Palmer, J. A. , Payne, A. M. , Mirdamadi, J. L. , Ting, L. H. , & Borich, M. R. (2025). Delayed cortical responses during reactive balance after stroke associated with slower kinetics and clinical balance dysfunction. Neurorehabilitation & Neural Repair, 39(1), 16–30. 10.1177/15459683241282786 PMC11723813 39328051

[tjp70847-bib-0059] Pollock, C. L. , Ivanova, T. D. , Hunt, M. A. , & Garland, S. J. (2014). Motor unit recruitment and firing rate in medial gastrocnemius muscles during external perturbations in standing in humans. Journal of Neurophysiology, 112(7), 1678–1684. 10.1152/jn.00063.2014 24990568

[tjp70847-bib-0060] Promsri, A. (2022). Modulation of bilateral lower‐limb muscle coordination when performing increasingly challenging balance exercises. Neuroscience letters, 767, 136299. 10.1016/j.neulet.2021.136299 34699944

[tjp70847-bib-0061] Tacca, N. , Levine, J. T. , Heimann, M. K. , Schlink, B. R. , Cady, S. , Colachis, S. C. , Baumgart, I. , Bollinger, A. , Dunlap, C. , Putnam, P. , Darrow, M. J. , Wengerd, L. , Pons, J. L. , Friedenberg, D. A. , & Meyers, E. C. (2026). Leveraging neural drive to assess hand motor function in individuals with chronic stroke. Journal of Neuroengineering and Rehabilitation, 23(1), 7. 10.1186/s12984-025-01806-6 PMC12781281 41507923

[tjp70847-bib-0062] Tamura, S. , Miyata, K. , Kobayashi, S. , Takeda, R. , & Iwamoto, H. (2022). The minimal clinically important difference in Berg Balance Scale scores among patients with early subacute stroke: A multicenter, retrospective, observational study. Topics in Stroke Rehabilitation, 29(6), 423–429. 10.1080/10749357.2021.1943800 34169808

[tjp70847-bib-0063] Thomas, C. K. , Butler, J. E. , & Zijdewind, I. (2002). Patterns of pathological firing in human motor units. Advances in Experimental Medicine and Biology, 508, 237–244. 10.1007/978-1-4615-0713-0_29 12171117

[tjp70847-bib-0064] Thompson, C. K. , Negro, F. , Johnson, M. D. , Holmes, M. R. , Mcpherson, L. M. , Powers, R. K. , Farina, D. , & Heckman, C. J. (2018). Robust and accurate decoding of motoneuron behaviour and prediction of the resulting force output. The Journal of Physiology, 596(14), 2643–2659. 10.1113/JP276153 PMC6046070 29726002

[tjp70847-bib-0065] Ting, L. H. (2007). Dimensional reduction in sensorimotor systems: A framework for understanding muscle coordination of posture. Progress in Brain Research, 165, 299–321. 10.1016/S0079-6123(06)65019-X PMC4121431 17925254

[tjp70847-bib-0066] Vieira, T. M. M. , Loram, I. D. , Muceli, S. , Merletti, R. , & Farina, D. (2012). Recruitment of motor units in the medial gastrocnemius muscle during human quiet standing: Is recruitment intermittent? What triggers recruitment? Journal of Neurophysiology, 107(2), 666–676. 10.1152/jn.00659.2011 PMC3349625 21994258

[tjp70847-bib-0067] Welch, T. D. J. , & Ting, L. H. (2014). Mechanisms of motor adaptation in reactive balance control. PLOS ONE, 9(5), e96440. 10.1371/journal.pone.0096440 PMC4014487 24810991

[tjp70847-bib-0068] West, S. G. , Finch, J. F. , & Curran, P. J. (1995). Structural equation models with nonnormal variables: Problems and remedies. In R. H. Hyole (Eds.), Structural Equation Modeling: Concepts, Issues, and Applications (pp. 56–75). Sage Publication, Inc Thousand Oaks.

[tjp70847-bib-0069] Winter, D. , Prince, F. , Frank, J. , Powell, C. , & Zabjek, K. (1996). Unified theory regarding A/P and M/L balance in quiet stance Journal of Neurophysiology, 75(6), 2334–2343. 10.1152/jn.1996.75.6.2334 8793746

[tjp70847-bib-0070] Winter, D. A. , Patla, A. E. , Ishac, M. , & Gage, W. H. (2003). Motor mechanisms of balance during quiet standing. Journal of Electromyography and Kinesiology : Official Journal of the International Society of Electrophysiological Kinesiology, 13(1), 49–56. 10.1016/s1050-6411(02)00085-8 12488086

[tjp70847-bib-0071] Winter, D. A. , Prince, F. , Stergiou, P. , & Powell, C. (1993). Medial‐lateral and anterior‐posterior motor responses associated with centre of pressure changes in quiet standing. Neuroscience Research Communications, 12, 141–148.

[tjp70847-bib-0072] Wutzke, C. J. , Mercer, V. S. , & Lewek, M. D. (2013). Influence of lower extremity sensory function on locomotor adaptation following stroke: A review. Topics in Stroke Rehabilitation, 20(3), 233–240. 10.1310/tsr2003-233 23841971

[tjp70847-bib-0073] Yamanaka, E. , Goto, R. , Kawakami, M. , Tateishi, T. , Kondo, K. , & Nojima, I. (2023). Intermuscular coherence during quiet standing in sub‐acute patients after stroke: An exploratory study. Brain Sciences, 13(12), 1640. 10.3390/brainsci13121640 PMC10742243 38137088

[tjp70847-bib-0074] Zaback, M. , Missen, K. J. , Adkin, A. L. , Chua, R. , Inglis, J. T. , & Carpenter, M. G. (2023). Cortical potentials time‐locked to discrete postural events during quiet standing are facilitated during postural threat exposure. The Journal of Physiology, 601(12), 2473–2492. 10.1113/JP284055 37060169

[tjp70847-bib-0075] Zaback, M. , & Thompson, C. K. (2026). Independent directional tuning of the human triceps surae muscles during standing postural control. Journal of Applied Physiology, 140(4), 846–861. 10.1152/japplphysiol.00626.2025 PMC13154211 41615326

