# Supplementary material for: Disrupted bilateral co‐ordination of soleus motor units during early subacute stroke rehabilitation
Source: J Physiol. 2026 Sep 18;604(20):8616–35. doi: 10.1113/JP290882 (PMC13674189; doi:10.1113/JP290882)
Supplement: Supplementary file 1 [file TJP-604-8616-s001.pdf]

The Journal of Physiology

Disrupted Bilateral Coordination of Soleus Motor Units during Early Subacute Stroke Rehabilitation

Jackson T Levine, Xin S Yu, Alyssa Jones, Rebecca Munoz, Martin Zaback, Christopher K Thompson, Dario Farina, Simon Avrillon, and José L. Pons
DOI: 10.1113/JP290882

Corresponding author(s): Jackson Levine (levinej@u.northwestern.edu)

The referees have opted to remain anonymous.

Review Timeline:

Submission Date:	07-Jan-2026
Editorial Decision:	17-Feb-2026
Revision Received:	03-Apr-2026
Editorial Decision:	29-Apr-2026
Revision Received:	28-Jul-2026
Accepted:	25-Aug-2026

Senior Editor: Richard Carson

Reviewing Editor: Mathew Piasecki

Transaction Report:

(Note: With the exception of the correction of typographical or spelling errors that could be a source of ambiguity, letters and reports are not edited. Depending on transfer agreements, referee reports obtained elsewhere may or may not be included in this compilation. Referee reports are anonymous unless the Referee chooses to sign their reports.)

Re: JP-RP-2026-290882 "Disrupted Bilateral Coordination of Soleus Motor Units during Early Subacute Stroke Rehabilitation" by Jackson T Levine, Xin S Yu, Alyssa Jones, Rebecca Munoz, Martin Zaback, Christopher K Thompson, Dario Farina, Simon Avrillon, and José L. Pons

Dear Dr Levine,

Thank you for submitting your manuscript to The Journal of Physiology. It has been assessed by a Reviewing Editor and by 2 expert referees and we are pleased to tell you that it is potentially acceptable for publication following satisfactory major revision.

Please advise your co-authors of this decision as soon as possible.

The referee reports are copied at the end of this email.

Please address all the points raised and incorporate all requested revisions or explain in your Response to Referees why a change has not been made. We hope you will find the comments helpful and that you will be able to return your revised manuscript within 2 months. If your article is NOT for a Special Issue, you may have 9 months to revise. If you require an extension, please contact journal staff: jp@physoc.org. Please note that this letter does not constitute a guarantee for acceptance of your revised manuscript.

Your revised manuscript should be submitted online using the link in your Author Tasks: Link Not Available. This link is accessible via your account as Corresponding Author; it is not available to your co-authors. If this presents a problem, please contact journal staff (jp@physoc.org). Image files from the previous version are retained on the system. Please ensure you replace or remove any files that are being revised.

If you do not wish to submit a revised version of your manuscript, you must inform our journal staff (jp@physoc.org) or reply to this email to request withdrawal. Please note that a manuscript must be formally withdrawn from the peer review process at one journal before it may be submitted to another journal.

TRANSPARENT PEER REVIEW POLICY: To improve the transparency of its peer review process, The Journal of Physiology publishes online as supporting information the peer review history of all articles accepted for publication. Readers will have access to decision letters, including Editors' comments and referee reports, for each version of the manuscript, as well as any author responses to peer review comments. Referees can decide whether or not they wish to be named on the peer review history document.

ABSTRACT FIGURES: Authors are expected to use The Journal's premium BioRender account to create/redraw their Abstract Figures. Information on how to access this account is here: https://physoc.onlinelibrary.wiley.com/journal/14697793/biorender-access.

This will enable Authors to create and download high-resolution figures. If authors have used the free BioRender service, they can use the instructions provided in the link above to download a high-resolution version suitable for publication.

The link provided should only be used for the purposes of this submission. Authors will be charged for figures created on this account if they are not related to this manuscript submission.

LANGUAGE EDITING AND SUPPORT FOR PUBLICATION: If you would like help with English language editing, or other article preparation support, Wiley Editing Services offers expert help, including English Language Editing, as well as translation, manuscript formatting, and figure formatting at www.wileyauthors.com/eeo/preparation. You can also find resources for Preparing Your Article for general guidance about writing and preparing your manuscript at www.wileyauthors.com/eeo/prepresources.

RIGOUR AND REPRODUCIBILITY: We recommend authors consult The Journal's Rigour and Reproducibility requirements to ensure that their manuscript is suitable for publication: https://physoc.onlinelibrary.wiley.com/pb-assets/hub-assets/physoc/documents/TJP-Rigour-and-Reproducibility-Requirements.pdf

REVISION CHECKLIST:

Check that your Methods section conforms to journal policy: https://jp.msubmit.net/cgi-bin/main.plex?form_type=display_requirements#methods.

Check that data presented conforms to the statistics policy: https://jp.msubmit.net/cgi-bin/main.plex?form_type=display_requirements#statistics.

Upload a full Response to Referees file. To create your 'Response to Referees': copy all the reports, including any comments from the Senior and Reviewing Editors, into a Microsoft Word, or similar, file and respond to each point, using font or background colour to distinguish comments and responses and upload as the required file type.

Please upload two versions of your manuscript text: one with all relevant changes highlighted and one clean version with no changes tracked. The manuscript file should include all tables and figure legends, but each figure/graph should be uploaded as separate, high-resolution files.

You may also upload:

- 'Potential Cover Art' for consideration as the issue's cover image
- Appropriate Supporting Information (Video, audio or data set: see https://jp.msubmit.net/cgi-bin/main.plex?form_type=display_requirements#supp).

We look forward to receiving your revised submission.

If you have any queries, please reply to this email and we will be pleased to advise.

Yours sincerely,

Richard Carson
Senior Editor
The Journal of Physiology

REQUIRED ITEMS

- Author photo and profile. First or joint first authors are asked to provide a short biography (no more than 100 words for one author or 150 words in total for joint first authors) and a portrait photograph. These should be uploaded and clearly labelled together in a Word document with the revised version of the manuscript. See Information for Authors for further details.

- The reference list must be in alphabetical order, rather than numbered, to comply with our Journal format.

- Your manuscript must include a complete Additional Information section, including competing interests; funding; author contributions and acknowledgements.

- Please upload separate high-quality figure files via the submission form.

- Please ensure that any tables are editable and in Word format, and wherever possible, embedded in the article file itself.

- Please ensure that the Article File you upload is a Word file.

- Please include an Abstract Figure file and an Abstract Figure legend. An appropriate figure legend, which should not exceed 150 words in length, should be included in the main manuscript file. The Abstract Figure is a piece of artwork designed to give readers an immediate understanding of the research and should summarise the main conclusions. If possible, the image should be easily 'readable' from left to right or top to bottom. It should show the physiological relevance of the manuscript so readers can assess the importance and content of its findings. Abstract Figures should not merely recapitulate other figures in the manuscript. Please try to keep the diagram as simple as possible and without superfluous information that may distract from the main conclusion(s). Abstract Figures must be provided by authors no later than the revised manuscript stage and should be uploaded as a separate file during online submission labelled as File Type 'Abstract Figure'. Please also ensure that you include the figure legend in the main article file. All Abstract Figures should be created using BioRender. Authors should use The Journal's premium BioRender account to export high-resolution images. Details on how to use and access the premium account are included as part of this email.

- The corresponding author must provide an institutional email address (not a personal address) for their author account. We

encourage ALL co-authors to also provide institutional email addresses. If this cannot be provided (as corresponding author), then a stamped letter must be provided from the institution which confirms their role and employment there (please upload this with the revised submission).

EDITOR COMMENTS

Reviewing Editor:
Your manuscript has been reviewed by two experts in this field. Although largely favourable, both offer several constructive and overlapping points that should be addressed before publication can be considered. Please pay particular attention to the suggestion of inclusion of appropriate effect sizes and a clearer description of the methods, as outlined by both reviewers.

Senior Editor:
Please give particular attention to the selection of statistical analysis methods. In this regard, it is critical that different sources of variance are demarcated appropriately, and not pooled in a manner such that within- and between-participant variance is conflated.

In respect of the suggestion that effect sizes be reported, please consider also reporting confidence intervals for the effect size estimates.

REFEREE COMMENTS

Referee #1:

Note that there were no page or line numbers in the manuscript so the authors will have to search to find some of the quotes below.

1. The authors are to be commended for a rigorous study employed in early sub-acute stroke. I appreciated knowing the time from stroke to first session. I would also like to know the time from admission to rehabilitation to first session - this would allow the reader to know how much intensive rehabilitation they had prior to testing.

2. Table 1 should include age.

3. Fig 1C - please clarify if these are EMG or MU PSTH waveforms being presented.

4. Fig 2 - Can you draw lines between the dots to show individual changes? Probably only possible for participants after stroke

5. Data analysis: COP - RMS COP DISPLACEMENT and COP speed were computed

6. I commend the authors for including participants who may have needed assistance in standing. However, the physiotherapy assistance (ratings of 4 or 5) would alter the COP data. We know that even touch assistance (rating of 3) will improve people's balance. And if a participant had less assistance in visit 2, the COP data get worse instead of better. I think mixing the data of those needing assistance with those that did not makes the data difficult to interpret. As an aside, I am surprised that someone with a BBS of 5 would be admitted to inpatient rehabilitation - this means that the person could probably sit unsupported for 2 min and maybe is a one-person transfer? Most rehab facilities won't accept someone who is a two-person transfer. If they can't stand for 30 s, then the PT was probably using assistance of 5 in testing. Combining this person, with the BBS of 48 is potentially problematic.

7. Your MU yield description need expansion. You were able to decompose an average of 10-13 MU per participant and task, which is great (especially for the females). But then you say, you only achieved this in 41 out of 112 conditions. Theoretically, then, most could have come from non-paretic muscles in visit 2. Can you describe in more details which tasks, legs, and visits your data come from? Yield is even lower in controls...

8. Given that some of your non-significant findings have p-values less than 0.1, you were likely underpowered for the wide variability across patients. I wonder if reporting effect sizes would help in the clinical interpretation. What was the correlation between BBS and assist level? I ask because the BBS variability could also be a random effect if they are not correlated. Do you have Fugl-Meyer scores for the affected limb? Would the extent of hemiparesis be affecting your results?

9. Fig 3.

• I am perplexed that the statistics did not reveal a difference in the anteroposterior direction. It looks like clear facilitation with anterior COP peaks and inhibition with posterior COP peaks.

• Are you plotting all individual data, including the outliers that were removed from statistics? This query applied to other figures as well.

• Also was there no difference between visit 1 and 2 in stroke group? One week of rehabilitation has no impact on your findings? You have a 10 point improvement in BBS over the week and this does not translate into any physiological improvements?

10. Fig 4.

• It is very difficult to tell the difference between Visit 1 and 2 and eyes open and closed with 4 lines in the same plot. Can you separate Visit or Task so there are only 2 conditions depecting in each plot? And the mean values overlap each other (perhaps that is your point) but can these be offset so each colour can be visualized?

• When plotting the individual data in the first row, can you specify whether this participant the affected was right or left side? I am trying to figure out if the shift clockwise in the affected side is related to side of paresis. Is "the greatest activation of the affected limb occurred during lateral shifts along the medio-lateral axis" because 9 of your participants had right hemiparesis and only 5 had hemiparesis?

• Also, It is known that when the affected leg is the dominant leg, there is more disability than when the affected leg is the nondominant leg. Did you ask participants about leg dominance?

• In the legend please indicate that Figure 4B top is PSTH and 4B bottom is EMG.

11. Fig 5.

• Could you expand the legend so it is more explanatory? There is importance to the color that is not described. Panel A (top row), the left panel is CST for right leg, middle panel is ipsilateral triggering from the right leg, right panel is contralateral triggering from the right leg onto the left leg. Do I have that right?

• Panel C. It is not easy to relate the narrative to the figure. You write "For PSTH, the proportion of significant contralateral-triggered amplitudes was 0.68 for control left to right, 0.66 for control right to left, 0.60 for unaffected to affected, and 0.72 for affected to unaffected." Presumably, task and visit are collapsed in this statement. In the figure, presumably task is collapsed but visit is now separated out? And why are there no individual data in Panel C? It is interesting that the values are so high for Visit 2 affected to unaffected.

• Fig 5D is not referred to in the narrative.

12. Fig 7

• If the data point in red did not exceed the criterion for outlier, then you should not label it as such.

• Visit 1 and Visit 2 are not independent of each other, as they are from the same participant. Wouldn't you be better off doing a repeated measures correlation in R that doesn't violate the assumptions of independence in the regression?

13. In the discussion, it is noted that the clinical outcomes improve but, with one exception, the physiological data do not. I think this outcome may be influenced by the assistance provided to the participants and by not differentiating between right and left hemiparesis (see my comments above).

14. In the discussion, the authors suggest that soleus could make up for hip weakness to "provide stabilizing torques along the mediolateral axis". This doesn't make sense to me, clinically, because typically people have more paresis distally than proximally. So the idea that a person following stroke would activate a distal muscle more to make up for proximal weakness would be a rare phenomenon in my experience.

15. The authors also suggest that "the lateral tuning could result from a pre-emptive strategy to cognitively avoid shifts toward the affected limb due to reduced balance confidence" (bolding is mine). I am not convinced that any pre-emptive strategy like this would be at the cognitive level.

16. The authors note that "PSTH absolute lag decreased from visit 1 to visit 2, marking the only observed longitudinal improvement in neural or balance metrics. This suggests that improved bilateral synchronization may be a critical component of balance recovery". While philosophically I don't disagree with the statement, I think the authors need to be cautious that they did a lot of comparisons in a small sample and one may have just happened to reach significance. Perhaps they could soften the implication statement.

Referee #2:

This is an interesting manuscript reporting on coordination of soleus motor units during quiet standing under different conditions in individuals who have suffered a stroke and neurologically normal controls. There are novel findings related to control of motor units in an important postural muscle in both legs in a task that is very easy for controls but can be very difficult for people who have suffered a stroke. The findings and discussion of potential mechanisms are of interest to neuroscientists and rehabilitation professionals. The study is well-designed but there are some concerns about robustness of some data due to high within and between subject variability of some measures. There are also some methodological issues that need clarification and some important findings that are not fully reported or discussed. Specific comments are provided below. Unfortunately, there were no page or line numbers provided in the pdf making it difficult to specify where in the document this review refers to for additions/corrections.

Key Points Summary

It is well-understood that unilateral strokes have effects on control of muscle activity and motion in both limbs. Thus, the terms "unaffected limb" and "affected limb" should be replaced with "less affected limb " and "more affected limb" here and throughout the document.

Introduction

This section is clearly written and provides excellent justification for this investigation.

Methods

This section is also clearly written but some methodological issues needed to be clarified.

1. A short description of the in-patient rehabilitation between week 1 and week 2 should be provided with some attention to differences of approach associated with baseline BBS scores.

2. Under "Bipolar EMG", the authors should describe the types of "significant artifacts" that were observed resulting in exclusion of data from the further analysis. Relatedly, the average and range of percentage of channels excluded on stroke patients and controls should be reported.

3. Figure 1 - I presume the COP vs time profile is for a-p motion but this is not specified and a calibration should also be provided. Similarly, calibration of the bipolar EMG signal (mV or µV) and PSTH (# of spikes in 5 ms bins) should be provided.

4. Under "Peristimulus waveforms and direction tuning" the terms medial and lateral should be defined for this task. Medial usually denotes towards body midline and lateral denotes away from body midline. Obviously, COP motion can cross body midline such that a motion in one direction can initially be medial and become lateral. Should sway towards right/left leg be used for controls and towards less affected/more affected leg for stroke patients?

Results

5. Under "Balance and center of pressure", it is important to describe that m-l sway amplitude and speed was similar to a-p amplitude and speed in stroke patients but was clearly smaller in the m-l direction in controls as would be expected. Possible causes of the high m-l sway amplitude and speed in stroke patients given the larger base of support in m-l than in a-p should be considered in the Discussion section.

6. Figure 2B: The ordinate scales for a-p and m-l COP metrics should be the same to ease readers' comparisons of a-p vs m-l sway.

7. Under "High-density EMG decomposition", the lower yield of motor units in stroke patients and the associated exclusion of 3 participants from the analysis are concerns that should be discussed in the Discussion section in relation to interpretation of the results for activation amplitudes during COP peaks. Notably, it appears that there were a number of statistical comparisons that approached significance but are not described. For example, sentences such as: "There were neither effects nor interactions in other directions ($P>0.061$)." and "... with no other differences between limbs ($P>0.057$). There were neither effects nor interactions in other direction ($P>0.086$)." The authors should consider whether some of the $p<0.1$ main effects or interactions warrant mention and discussion given the relatively small groups and high inter-subject variability of some data. This can also be considered in a Discussion of limitations of this research.

8. Figure 3: What exactly is meant by "Area under the curve between -0.4s to 0s relative to the peak." in relation to the ordinate scales for PSTH amplitude in A and B? How was the time from -0.4s to 0s determined to be the appropriate time-period to evaluate? What exactly are the plotted numbers of the ordinate scales for PSTH amplitude in A and B as they are quite different but but have the same u nits (pulses/peak). Also, are these data in a and B from eyes open or closed conditions?

9. Under "Directional tuning", the variability of mean angles among subjects in both groups for each leg is very high for both PSTH and EMG data. For example, control right and left limbs for PSTH had mean +/- SDs of 0.79 o +/- 40.62o and 6.54 o +/- 37.56 o. Indeed, according to Fig. 4B absolute differences of mean angles of the two legs exceeded 90o in several control subjects. How similar (reliable) were the mean angles for each leg and the absolute differences between legs in visit 1 and visit 2 in controls? Is it reasonable to assume that the absolute difference between angles for the two legs signifies a real difference? This issue should be discussed as a limitation in the interpretation of directional tuning data. Finally, EMG amplitude is not normalized, yet across-group comparisons are made. Of course, normalization is difficult in stroke patients but the lack of normalization should be discussed in a Discussion section on limitations.

10. Figure 4A: The control left and unaffected leg tuning curves should be plotted in brighter colors as they are difficult to see.

11. Figure 5: The legend next to C is, I think, actually the legend for D. Also, are the examples in A and B from eyes open or closed conditions and from visit 1 or 2?

12. Under "Cross correlation of motor unit...", it is stated: "Controls demonstrated greater correlation coefficients (0.79{plus minus}0.19) compared to the stroke group (0.68{plus minus}0.17)." However, no p-value for the statistical comparison of the correlation coefficients is provided, thus it is not clear that the coefficients were statistically larger in controls.

13. Under "Forward regression ...", it is stated: "Data was pooled across tasks as task effects were minimal." However, this pooling of data would provide multiple data points for each subject whereas such regressions would normally be performed using a single data point for each subject. Using two data points from each subject would violate statistical assumptions of independence for each data point. Figure 7 has 22 data points, indicating there were two from each of the 11 stroke subjects who had PSTH neuromuscular data. It would be more appropriate to plot the data with different symbols for eyes-open vs closed conditions and perform separate regression for those conditions. Also, it should be specified whether BBS and neuromuscular data was from visit 1 or visit 2 was used in Fig. 7. Relatedly, it would be interesting to provide a similar graph and regression coefficients for visit 2 given the improvement of BBS scores in visit 2.

Discussion

Overall, this section is clearly written and provides reasonable interpretations of the findiings. However, as mentioned above, there are some issues that should be addressed in a discussion section on limitations related to interpretations of these data.

END OF COMMENTS

Dear Dr. Carson,
Thank you for the helpful review of our manuscript. We have addressed all the reviewers' comments in the point-by-point response below.

REVIEWS
EDITOR COMMENTS

Reviewing Editor:
Your manuscript has been reviewed by two experts in this field. Although largely favourable, both offer several constructive and overlapping points that should be addressed before publication can be considered. Please pay particular attention to the suggestion of inclusion of appropriate effect sizes and a clearer description of the methods, as outlined by both reviewers.
We thank the editors and reviewers for their positive appreciation of our work. We have followed most of your suggestions, particularly on the statistics. You will find our responses below.

Senior Editor:
Please give particular attention to the selection of statistical analysis methods. In this regard, it is critical that different sources of variance are demarcated appropriately, and not pooled in a manner such that within- and between-participant variance is conflated.
We thank the editor for their comment. We have clarified the random structure of our linear mixed effect models to account for both random slopes and intercepts. The description of the models has been updated in the section Statistics of the methods as follow:

(P. 13; L. 312-317): "*We compared the amplitude of PSTH and EMG independently for each direction, as was done with the COP metrics, with Limb (control right, control left, paretic, non-paretic), Visit, and Task as fixed effects and Participant and Assist level as random effects. The random structure of all models included random slopes to also account for between participant variability in the fixed effects. Ultimately, the model associated with the lowest Bayesian information criterion was retained for the analysis.* ".

In respect of the suggestion that effect sizes be reported, please consider also reporting confidence intervals for the effect size estimates.
We have now included effect sizes and confidence intervals in the results section.

REFEREE COMMENTS

Referee #1:
Note that there were no page or line numbers in the manuscript so the authors will have to search to find some of the quotes below.
The line numbers have been added to the revised version of the manuscript. We will mention them next to our responses to your comments such that you can easily reach the section of interest in the manuscript.

1. The authors are to be commended for a rigorous study employed in early sub-acute stroke. I appreciated knowing the time from stroke to first session. I would also like to know the time from admission to rehabilitation to the first session - this would allow the reader to know how much intensive rehabilitation they had prior to testing.

This information has been added to Table 1 (P. 5). The average time from stroke to admission was 11±7 days and the time from stroke to session 1 was 19±8 days, with an average difference of 9±6 days between admissions and testing. This delay was mainly dedicated to informing patients and their families about the goal of the study, completing inclusion, and scheduling the testing sessions between regular rehabilitation sessions.

2. Table 1 should include age.

This information has been added to Table 1 (P. 6-7).

**Stroke Participant**	**Age (yrs)**	**Sex**	**Stroke Type**	**Stroke Location**	**Hemi Side**	**Time Stroke to Admission (Days)**	**Time Stroke to Session 1 (Days)**	**Baseline BBS (out of 56)**
1	62.0	Male	Ischemic	MCA	Left	16	24	48
2	75.7	Female	Ischemic	MCA	Right	6	18	28
3	71.1	Male	Ischemic	Thalamus	Right	4	11	34
4	56.6	Female	Hemorrhagic	BG	Left	10	15	29
5	19.0	Male	Hemorrhagic	FP	Left	10	15	5
6	75.8	Male	Ischemic	PCA	Right	4	10	30
7	67.2	Female	Ischemic	MCA	Right	17	26	37
8	65.8	Male	Ischemic + Hemorrhagic	MCA	Right	7	35	24
9	58.9	Female	Hemorrhagic	PLIC	Right	5	10	19
10	55.3	Female	Hemorrhagic	BG	Left	20	27	20
11	78.9	Male	Ischemic	FP	Right	5	12	34
12	55.4	Male	Ischemic + Hemorrhagic	MCA	Right	12	26	43
13	37.9	Female	Ischemic	MCA	Right	28	30	38
14	63.1	Female	Ischemic	PLIC	Left	9	13	9
**Avg ± Std**	**60.2 ±15.9**	**M/F 7/7**			**R/L 9/5**	**10.93 ±7**	**19.00 ±8**	**28.00 ±12**

Abbreviations: Middle Cerebral Artery (MCA), Posterior Cerebral Artery (PCA), Posterior Limb of Internal Capsule (PLIC), Basal Ganglia (BG), Fronto-parietal (FP).

3. Fig 1C - please clarify if these are EMG or MU PSTH waveforms being presented.

Fig 1C represents EMG waveforms. This information has been added in the figure and in the caption:

(P. 12; L. 294-297) "*C) The time-locked waveform averages (EMG shown in the figure) were computed during COP peaks in the anterior, posterior, medial, and lateral directions within the transverse plane of axial rotation, as well as in 5° increments (72 directions in total) around the participant.*"

4. Fig 2 - Can you draw lines between the dots to show individual changes? Probably only possible for participants after stroke

The figure has been updated (P. 15).

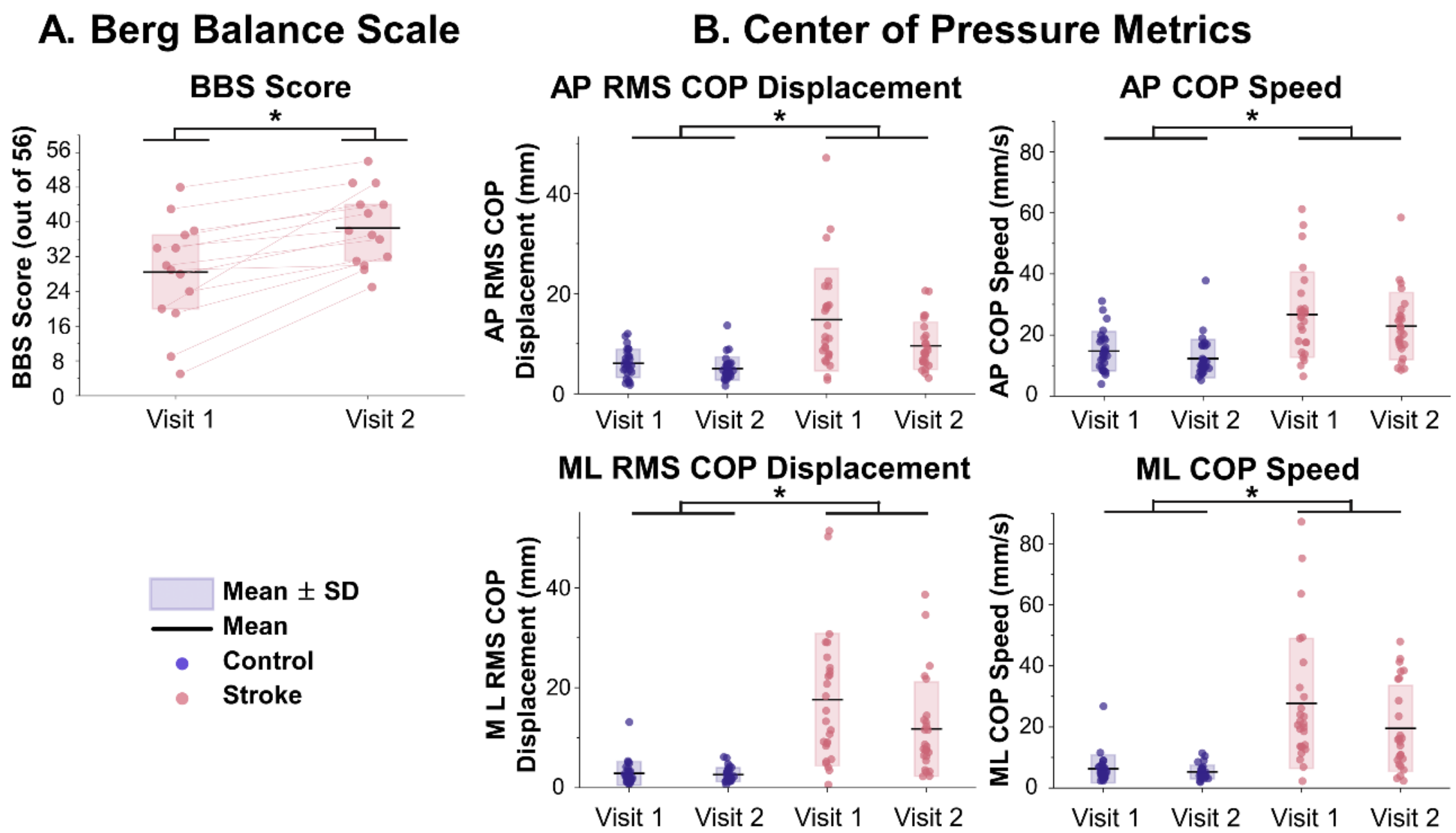

5. Data analysis: COP - RMS COP DISPLACEMENT and COP speed were computed

The term *displacement* has been added in the appropriate sections.

(P. 8; L.180-183): "*The **COP displacement** was computed along the medio-lateral and anterior-posterior axes from the split-belt treadmill and low pass filtered with a third order Butterworth filter at 5Hz. Root mean square (RMS) COP displacement and COP speed were computed for the anterior-posterior and medio-lateral directions as measures of balance variability and total displacement.*"

(P.13; L.307-312): "*We compared the **RMS COP displacement** (independently for different directions), COP speed (independently for different directions), absolute value of difference in circular mean angle between limbs, and cross correlation coefficient and lag using a linear mixed effects model with Group (control, patient), Visit and Task (eyes open, eyes closed) as fixed effects and Participant and Assist level (scored 0-5) as random effects to account for the degree of independence in the tasks.*"

(P.14; L.345-348): "***RMS COP displacement** in the anterior-posterior direction was greater in the stroke group (13.48±6.44mm) compared to controls (5.53±2.60mm, P<0.001, d=0.960, 95% CI: [0.565, 1.355]) and lower at visit 2 (7.14±4.27mm) compared to visit 1 (10.27±8.53mm, P=0.003[JTL1] , d=0.414, 95% CI: [0.004, 0.793]).* "

(P.14; L.352-355): "***RMS COP displacement** in the medio-lateral direction was greater in the stroke group (14.68±11.77mm) than controls (2.74±1.92mm, P<0.001, d=1.365, 95% CI: [0.949, 1.780]), and greater with eyes open (9.37±10.79mm) than eyes closed (7.40±9.43mm, P=0.009[JTL1] , d=1.693, 95% CI: [1.257, 2.128]).*"

6. I commend the authors for including participants who may have needed assistance in standing. However, the physiotherapy assistance (ratings of 4 or 5) would alter the COP data. We know that even touch assistance (rating of 3) will improve people's balance. And if a participant had less assistance in visit 2, the COP data get worse instead of better. I think mixing the data of those needing assistance with those that did not makes the data difficult to interpret. As an aside, I am surprised that someone with a BBS of 5 would be admitted to inpatient rehabilitation - this means that the person could probably sit unsupported for 2 min and maybe is a one-person transfer? Most rehab facilities won't accept someone who is a two-person transfer. If they can't stand for 30 s, then the PT was probably using assistance of 5 in testing. Combining this person with the BBS of 48 is potentially problematic.
We recognize that the physical therapist's assistance may have an effect on the balance control and, subsequently, on our measurements. For that reason, we included the level of assistance as a random effect in all models (except for the directional tuning because the circular statistics did not allow for it). We clarified the structure of our linear mixed effect models, including the description of random slopes to account for the variability of patient's responses between visits. In this way, we account for interindividual differences in patients' responses between visits while accounting for the effect of assistance on the data.

We added a sentence to our discussion to highlight the potential effect of assistance on our results.

(P.24; L.560-564): "*No other longitudinal changes were observed, which may be the result of the small sample size and the large variability between participants. The physical therapist assistance may have also impacted the outcomes, but this was accounted for in the statistical tests and the level of assistance did not change over time (average change: -0.43±0.63 points out of 5, P=0.251).*"

7. Your MU yield description need expansion. You were able to decompose an average of 10-13 MU per participant and task, which is great (especially for the females). But then you say, you only achieved this in 41 out of 112 conditions. Theoretically, then, most could have come from non-paretic muscles in visit 2. Can you describe in more details which tasks, legs, and visits your data come from? Yield is even lower in controls…

To improve the clarity of motor unit yield, we included the number of motor units extracted from each visit, task, and limb in Table 2. We excluded one control participant and three stroke participants due to the inability to extract any motor units from these individuals.

(P.15, L.364-367): "*Motor unit yields are shown in Table 2. We excluded one control participant and three stroke participants from the motor unit analyses because no motor units were identified for any muscle.*"

Table 2: Motor unit yield across limbs, groups and visits

		Stroke		Control	
		**Paretic**	**Non-Paretic**	**Right**	**Left**
**Eyes Closed**	**Visit 1**	8.8±10.2	5.0±6.4	11.1±10.7	9.1±9.1
	**Visit 2**	6.1±6.9	8.7±8.0	11.9±8.8	12.8±8.4
**Eyes Open**	**Visit 1**	9.2±8.2	5.1±5.6	11.0±11.2	8.3±8.1
	**Visit 2**	6.4±7.0	7.0±7.2	13.6±9.2	12.3±7.4

We also discuss motor unit yield in the last section:
(P.25-26, L.609-619): "*Thus, PSTH-derived metrics may be considered as a biomarker when assessing patients' control of balance. However, the application of such approaches is currently limited to high quality recordings capable of extracting multiple motor unit firings in patients. For example, it is noteworthy that our regression analysis only included individuals with motor units extracted in both visits, with three individuals in the stroke group excluded due to the inability to extract any motor units. The absence of extracted motor units may be explained by physiological factors, such as low muscle activation or reduced neural drive, but also poor signal quality, which are particularly present after stroke. Therefore, to extend the applicability of motor unit-based clinical analyses, future work must continue to improve HD-EMG sensors, extend investigations to intramuscular EMG, and enhance decomposition techniques to increase motor unit yields across wider populations.*"

8. Given that some of your non-significant findings have p-values less than 0.1, you were likely underpowered for the wide variability across patients. I wonder if reporting effect sizes would help in the clinical interpretation.
We thank the reviewer for their comments. We have now incorporated effect sizes and confidence intervals into the manuscript.

However, Gandevia & Heroux (2019, J Physiol) outlined guidelines for statistical analysis and interpretation for Journal of Physiology. They suggested caution against interpreting p values above 0.05. Therefore, in line with the journal guidelines, we refrained from interpreting these values or highlighting these p values to ensure we did not implicate their meaning in our discussion.

Gandevia, Simon, and Martin Héroux. "Statistics and Spin: It's Time to Improve." The Journal of Physiology [England], vol. 597, no. 16, August 2019, pp. 4411–12, https://doi.org/10.1113/JP278335.

What was the correlation between BBS and assist level? I ask because the BBS variability could also be a random effect if they are not correlated.

Below is a plot of the assist level versus the BBS score, with data pooled across visits and tasks. However, we could not include this as a random effect because we do not have the BBS scores for the control participants.

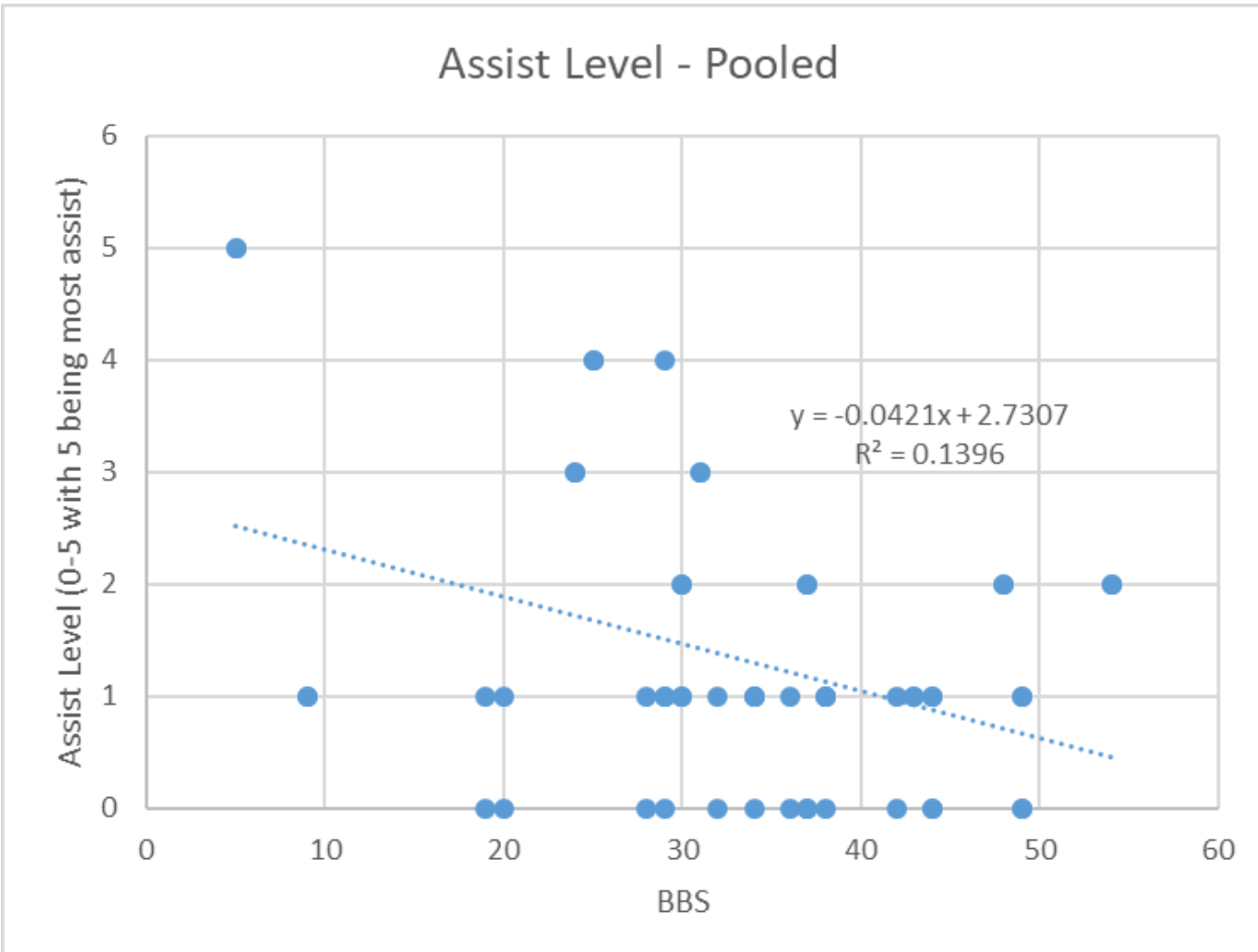

Do you have Fugl-Meyer scores for the affected limb? Would the extent of hemiparesis be affecting your results?
We did not evaluate Fugl-Meyer scores in this study.

9. Fig 3.

• I am perplexed that the statistics did not reveal a difference in the anteroposterior direction. It looks like clear facilitation with anterior COP peaks and inhibition with posterior COP peaks.
To minimize the number of effects tested, we did not assess the difference between directions in this analysis. We only focused on the differences between visits and between limbs in controls and patients. This analysis was performed separately for each direction.

• Are you plotting all individual data, including the outliers that were removed from statistics? This query applied to other figures as well.
All individual data is plotted except for outliers that were removed from statistics (values exceeding 3 standard deviations from the mean). This applies to all figures.

• Also was there no difference between visit 1 and 2 in stroke group? One week of rehabilitation has no impact on your findings? You have a 10 point improvement in BBS over the week and this does not translate into any physiological improvements?
COP displacement and speed decreased between visits. The only significant improvement over time that was observed in the neural metrics was the change in cross correlation lag of the PSTH. Otherwise, there were no other significant changes between visits for directional tuning and contralateral triggering. There were individual improvements in some of these metrics, but it is likely that the small sample size and variability between participants limited statistical power.

These were addressed in the following statement:
(P. 24, L.560-562): "*No other longitudinal changes were observed, which may be the result of the small sample size and the large variability between participants*."

Another factor that may have impacted the outcomes is the tracking of motor units between visits. Indeed, we may have identified slightly different subsets of motor units within the pool between visits, the decomposition algorithm being biased toward the largest active motor units. Although this does not impact the main outcomes of the study, this may have limited the sensibility of our measurements related to spatial recruitment and rate coding over time.

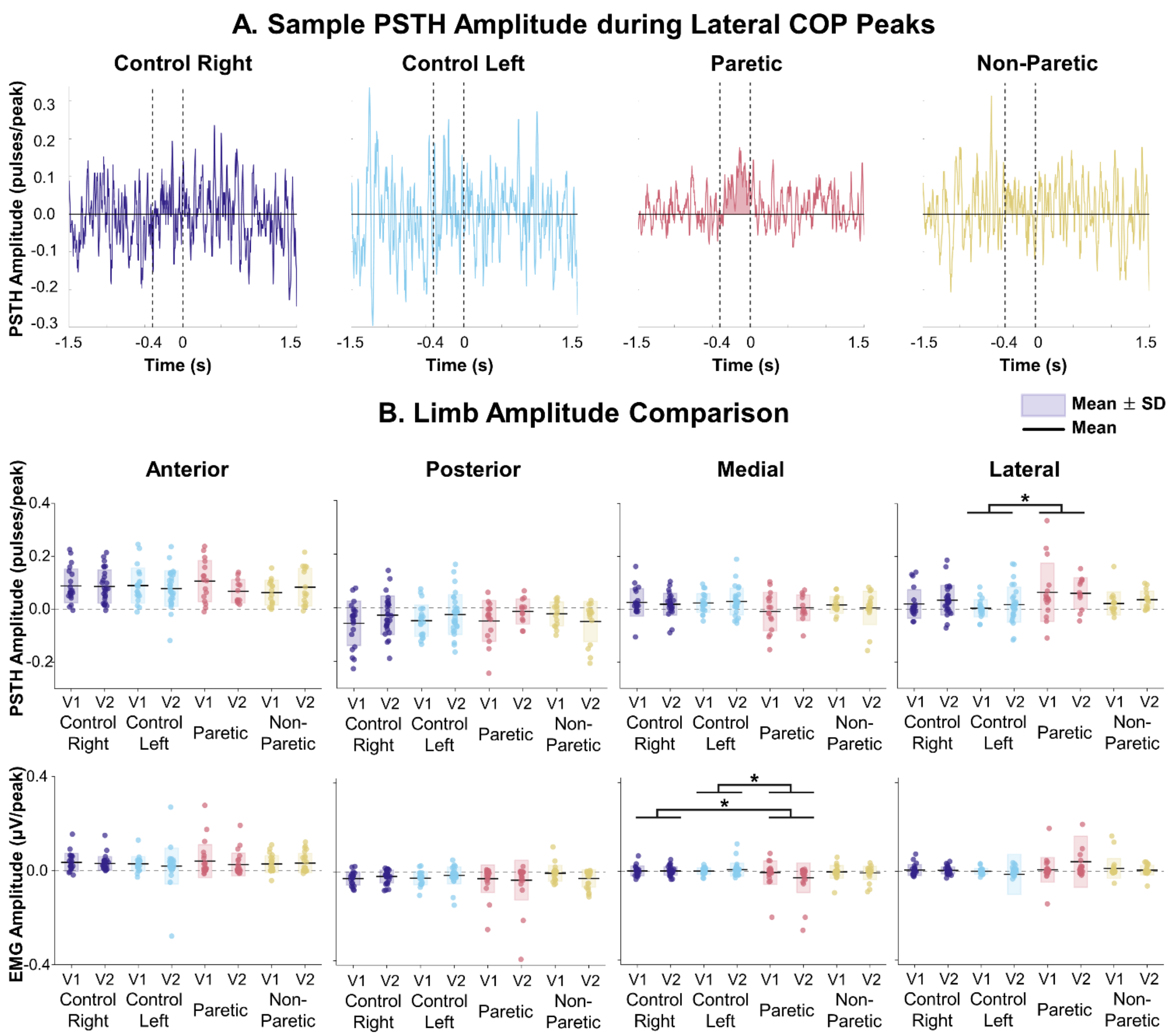

10. Fig 4.

• It is very difficult to tell the difference between Visit 1 and 2 and eyes open and closed with 4 lines in the same plot. Can you separate Visit or Task so there are only 2 conditions depecting in each plot? And the mean values overlap each other (perhaps that is your point) but can these be offset so each colour can be visualized?

To simplify this figure, the task identifier has been removed, as there was very minimal difference in tasks for any outcome measures. Now this simply shows the difference between visit 1 and 2 with a dashed versus solid line. We also offset the mean values as you suggested to better highlight the comparison and made all lines thicker to make it clearer. (P.18; L.414):

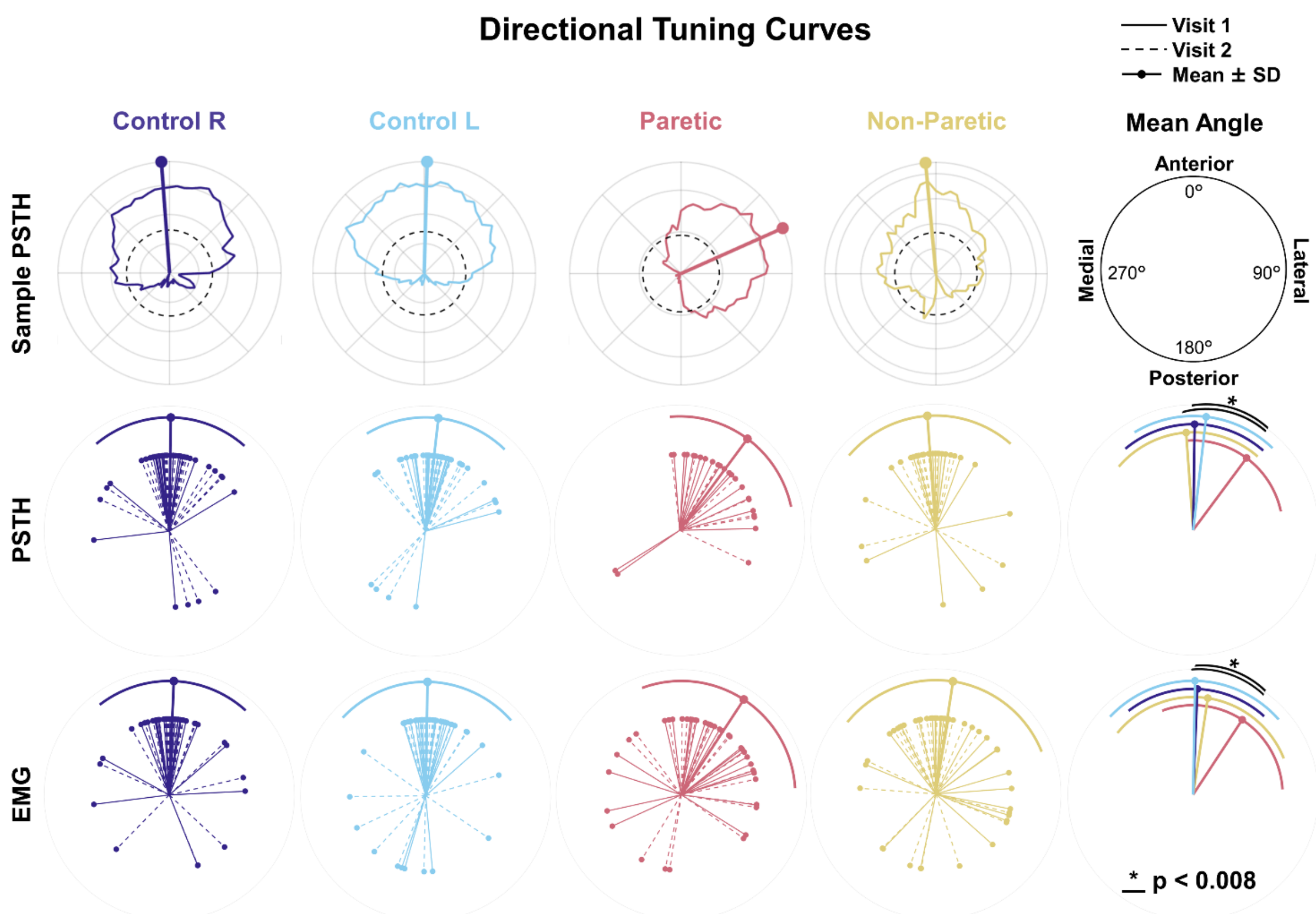

• When plotting the individual data in the first row, can you specify whether this participant the affected was right or left side? I am trying to figure out if the shift clockwise in the affected side is related to side of paresis. Is "the greatest activation of the affected limb occurred during lateral shifts along the medio-lateral axis" because 9 of your participants had right hemiparesis and only 5 had hemiparesis?

The side of the paresis does not affect the visualization of the results. We clarified this in the methods:

(P.10; L.246-250) "*To enable comparison between left and right limbs without introducing side bias, directional tuning curves from the left limb were reflected across the anterior–posterior*

axis. This transformation aligned both limbs to a common coordinate system such that anterior = 0°, posterior = 180°, medial = 270°, and lateral = 90° regardless of limb side (Fig. 1)."

• Also, It is known that when the affected leg is the dominant leg, there is more disability than when the affected leg is the nondominant leg. Did you ask participants about leg dominance?
Unfortunately, leg dominance was not recorded in this study.

• In the legend please indicate that Figure 4B top is PSTH and 4B bottom is EMG.
This analysis was removed, as it presented redundant information to focus on the main differences in the directional tuning curves.

11. Fig 5.

• Could you expand the legend so it is more explanatory? There is importance to the color that is not described. Panel A (top row), the left panel is CST for right leg, middle panel is ipsilateral triggering from the right leg, right panel is contralateral triggering from the right leg onto the left leg. Do I have that right?

You are correct in your interpretation.
We have updated the caption of the figure to include this:
(P.19-20; L.439-446) "A) Control right (purple) and left (blue) and B) stroke participant paretic (red) and non-paretic (yellow) ipsilateral- and contralateral-triggered peristimulus time histogram (PSTH) waveforms while standing with eyes closed (visit 2 for the control and visit 1 for the stroke participant). *As a guide to read these figures, for the top row of Panel A, the left column is the CST of the control right limb with peaks identified. The middle column is the ipsilateral activation or activation amplitude of the right limb during right limb peaks in CST. The left column is the contralateral activation or activation amplitude of the left limb during right limb peaks in CST.* "

• Panel C. It is not easy to relate the narrative to the figure. You write "For PSTH, the proportion of significant contralateral-triggered amplitudes was 0.68 for control left to right, 0.66 for control right to left, 0.60 for unaffected to affected, and 0.72 for affected to unaffected." Presumably, task and visit are collapsed in this statement. In the figure, presumably task is collapsed but visit is now separated out? And why are there no individual data in Panel C? It is interesting that the values are so high for Visit 2 affected to unaffected.
You are correct that task and visit were collapsed in the ratios presented in the text while they were separated by visits in the figure. We ultimately decided to remove this analysis as stated above.

• Fig 5D is not referred to in the narrative.
We have updated the figure references as follows (referring to the Fig 5C as we removed analysis in former C):
(P.18, L.423-426): "*We then calculated the difference between contralateral- and ipsilateral-triggered activation amplitudes: zero indicates equal activation, and negative values indicate*

*reduced activation of the limb when peaks of activity are observed in the contralateral limb (**Fig. 5C**)"*

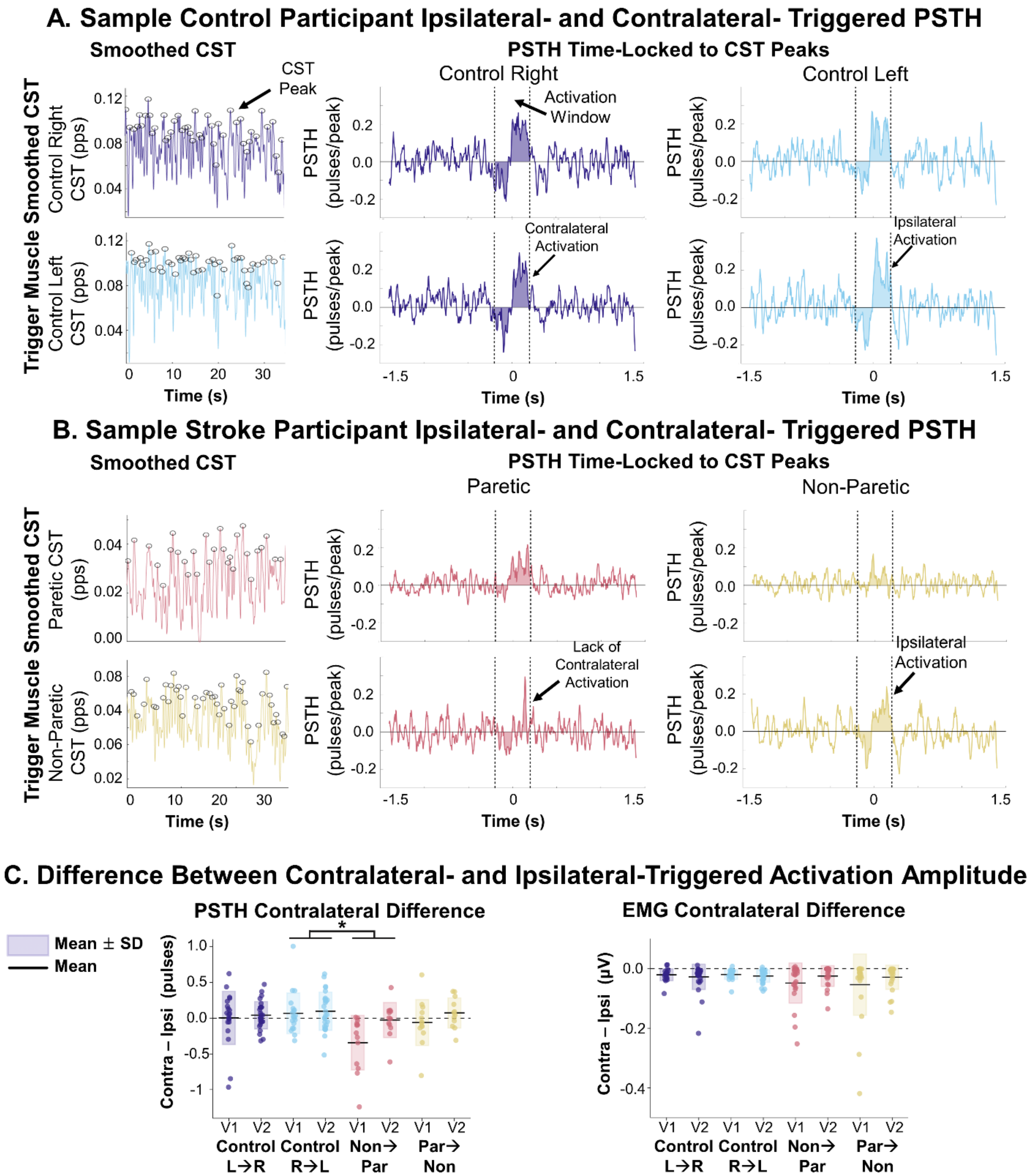

12. Fig 7

• If the data point in red did not exceed the criterion for outlier, then you should not label it as such.

We removed this statement.

• Visit 1 and Visit 2 are not independent of each other, as they are from the same participant. Wouldn't you be better off doing a repeated measures correlation in R that doesn't violate the assumptions of independence in the regression?
Thank you for highlighting this error. This was also pointed out by the other reviewer. Following your advice, we have pooled the data per participant and ran two analyses. First, we have performed a linear regression between delta lag and delta Berg Balance scale score between visits, showing a negative significant correlation between both variables ($r = -0.94$; $P < 0.001$). Second, as you suggested, we ran a repeated measures correlation to further confirm this result ($r = -0.78$; $P = 0.002$). The results have been updated to report these analyses:

(P.21-22; L. 479-487) "*Only PSTH absolute lag demonstrated a significant longitudinal change for stroke participants. Therefore, the relationship between the change in PSTH absolute lag and the change in functional balance score (i.e., BBS score) for stroke participants was assessed via linear regressions. Data was averaged across tasks per participant as task effect was absent. A linear regression between the changes in lag between visits and the change in BBS score between visits revealed a significant negative correlation ($r=-0.94$, $P<0.001$). This was further confirmed by a repeated measures correlation analysis with a significant negative correlation between both variables ($r = -0.78$; $p = 0.002$). Thus, an increase in BBS score was accompanied by a decrease in lag (or an increase in synchronization).*"

13. In the discussion, it is noted that the clinical outcomes improve but, with one exception, the physiological data do not. I think this outcome may be influenced by the assistance provided to the participants and by not differentiating between right and left hemiparesis (see my comments above).
As stated above, the only significant improvement over time that was observed in the neural metrics was the change in cross correlation lag of the PSTH. There were individual improvements in some of these metrics, but it is likely that the small sample size, and the variability between participants limited statistical power. The level of assistance was accounted for in our statistical models. Another factor we proposed was the identification of different subsets of motor units between visits.

Also, please note our response above on the interpretation of directional tuning of soleus activities in paretic and non-paretic limbs.

14. In the discussion, the authors suggest that soleus could make up for hip weakness to "provide stabilizing torques along the mediolateral axis". This doesn't make sense to me, clinically, because typically people have more paresis distally than proximally. So the idea that a person following stroke would activate a distal muscle more to make up for proximal weakness would be a rare phenomenon in my experience.
This was removed.

15. The authors also suggest that "the lateral tuning could result from a pre-emptive strategy to cognitively avoid shifts toward the affected limb due to reduced balance confidence" (bolding is mine). I am not convinced that any pre-emptive strategy like this would be at the cognitive level.
This was removed.

16. The authors note that "PSTH absolute lag decreased from visit 1 to visit 2, marking the only observed longitudinal improvement in neural or balance metrics. This suggests that improved bilateral synchronization may be a critical component of balance recovery". While philosophically I don't disagree with the statement, I think the authors need to be cautious that they did a lot of comparisons in a small sample and one may have just happened to reach significance. Perhaps they could soften the implication statement.
You are absolutely right. We have softened the implications of this statement:
(P.24; L.565-567): "*This change in PSTH absolute lag suggests a link between improvements in bilateral synchronization and balance recovery during early rehabilitation, but future studies with larger sample sizes will be necessary to corroborate these initial findings.*"

Referee #2:

This is an interesting manuscript reporting on coordination of soleus motor units during quiet standing under different conditions in individuals who have suffered a stroke and neurologically normal controls. There are novel findings related to control of motor units in an important postural muscle in both legs in a task that is very easy for controls but can be very difficult for people who have suffered a stroke. The findings and discussion of potential mechanisms are of interest to neuroscientists and rehabilitation professionals. The study is well-designed but there are some concerns about robustness of some data due to high within and between subject variability of some measures. There are also some methodological issues that need clarification and some important findings that are not fully reported or discussed. Specific comments are provided below. Unfortunately, there were no page or line numbers provided in the pdf making it difficult to specify where in the document this review refers to for additions/corrections.

Key Points Summary

It is well-understood that unilateral strokes have effects on control of muscle activity and motion in both limbs. Thus, the terms "unaffected limb" and "affected limb" should be replaced with "less affected limb " and "more affected limb" here and throughout the document.
You are absolutely correct that both limbs are affected after a stroke. To not use the term *unaffected*, we have instead used the terms *paretic* and *non-paretic* through the manuscript.

Introduction

This section is clearly written and provides excellent justification for this investigation.

Thank you for the positive appreciation of our work.

Methods

This section is also clearly written but some methodological issues needed to be clarified.

1. A short description of the in-patient rehabilitation between week 1 and week 2 should be provided with some attention to differences of approach associated with baseline BBS scores.
We have added a description of the in-patient rehabilitation in this section:
(P.6, L.147-150) “*All patients received standard physical therapy throughout the study as dictated by their clinicians. This consisted of a standard of 180 minutes of skilled therapy per day for five to six days per week, distributed between physical therapy (with a focus on high-intensity gait training), occupational therapy, and speech therapy*”

2. Under "Bipolar EMG", the authors should describe the types of "significant artifacts" that were observed resulting in exclusion of data from the further analysis. Relatedly, the average and range of percentage of channels excluded on stroke patients and controls should be reported.
We have now specified the types of artifacts that resulted in exclusion and the percentage of channels that were excluded on average for control and stroke participants:
(P.8; L.185-188): “*EMG signals from each channel were visually inspected offline. Channels with significant artifacts due to movements or disrupted electrode-skin contact were manually excluded from further analyses. Across control and stroke participants, an average of 1.5 and 1.9 channels out of 64 were removed, respectively.* ”

3. Figure 1 - I presume the COP vs time profile is for a-p motion but this is not specified and a calibration should also be provided. Similarly, calibration of the bipolar EMG signal (mV or µV) and PSTH (# of spikes in 5 ms bins) should be provided.
You are correct that this is an example of an AP COP profile. This has been specified in the figure as well as in the caption. The amplitude for these curves has been added.

(P.12, L.289-291) “A) Anteroposterior (AP) center of pressure (COP), bipolar EMG signal, and decomposed motor unit (MU) firings were extracted during a 30s trial where participants stood on two force plates during quiet standing (as shown in C).”

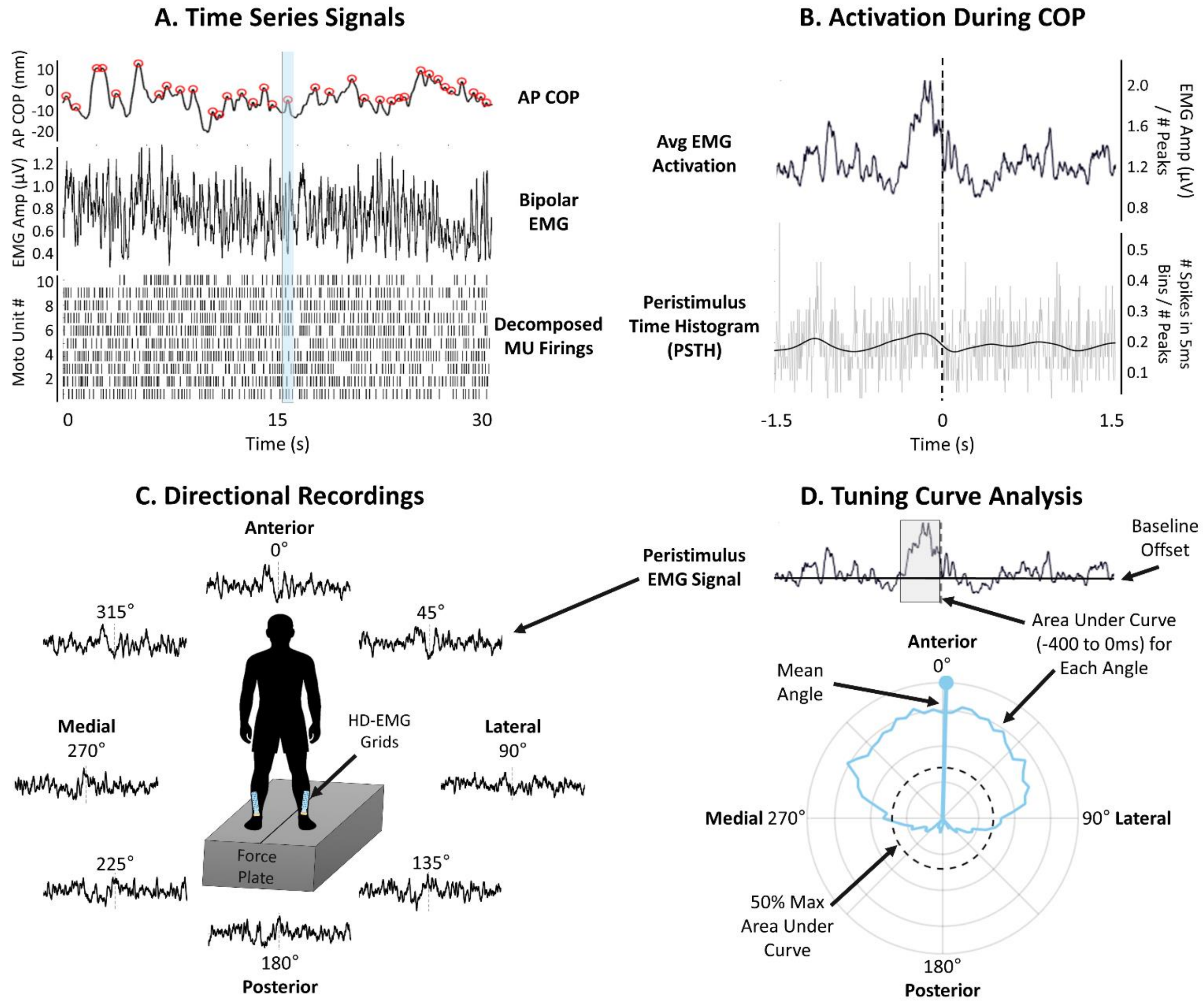

4. Under "Peristimulus waveforms and direction tuning" the terms medial and lateral should be defined for this task. Medial usually denotes towards body midline and lateral denotes away from body midline. Obviously, COP motion can cross body midline such that a motion in one direction can initially be medial and become lateral. Should sway towards right/left leg be used for controls and towards less affected/more affected leg for stroke patients?

We added the description in this section:

(P.10; L.246-253): "*To enable comparison between left and right limbs without introducing side bias, directional tuning curves from the left limb were reflected across the anterior–posterior axis. This transformation aligned both limbs to a common coordinate system such that anterior = 0°, posterior = 180°, medial = 270°, and lateral = 90° regardless of limb side (Fig. 1). A medial COP peak was defined as a peak directed toward the contralateral limb, whereas a lateral COP peak was defined as a peak directed away from the contralateral limb (i.e., toward the side of the limb of interest).*"

Results

5. Under "Balance and center of pressure", it is important to describe that m-l sway amplitude and speed was similar to a-p amplitude and speed in stroke patients but was clearly smaller in the m-l direction in controls as would be expected. Possible causes of the high m-l sway amplitude and speed in stroke patients given the larger base of support in m-l than in a-p should be considered in the Discussion section.

To minimize the number of effects tested, we did not assess the difference between directions in this analysis. We only focused on the differences between visits and between limbs in controls and patients. This analysis was performed separately for each direction.

6. Figure 2B: The ordinate scales for a-p and m-l COP metrics should be the same to ease readers' comparisons of a-p vs m-l sway.

We have updated the axes scale in the figure (P.15; L.360):

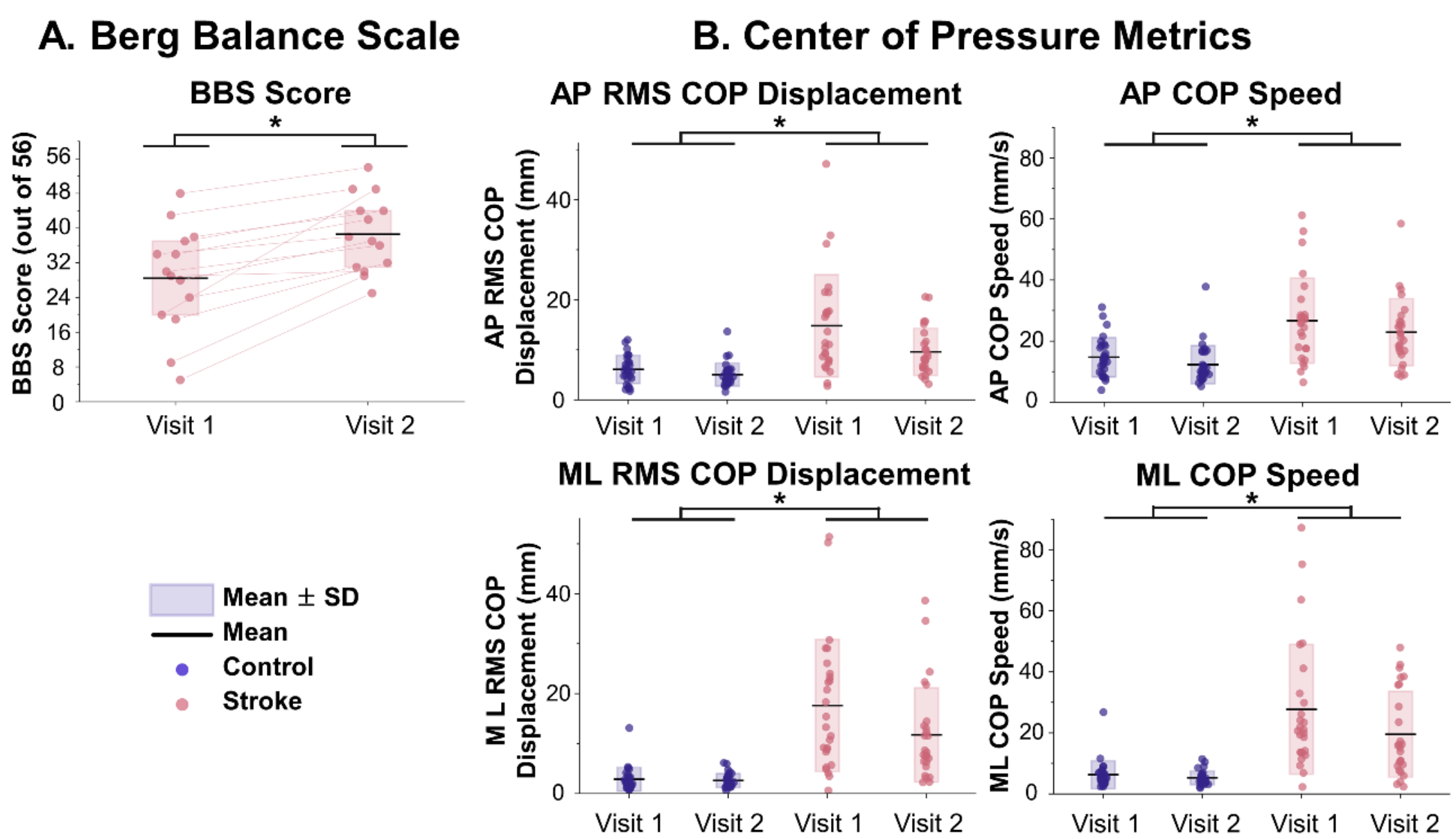

7. Under "High-density EMG decomposition", the lower yield of motor units in stroke patients and the associated exclusion of 3 participants from the analysis are concerns that should be discussed in the Discussion section in relation to interpretation of the results for activation amplitudes during COP peaks. Notably, it appears that there were a number of statistical comparisons that approached significance but are not described. For example, sentences such as: "There were neither effects nor interactions in other directions (P>0.061)." and "... with no other differences between limbs (P>0.057). There were neither effects nor interactions in other direction (P>0.086)." The authors should consider whether some of the p<0.1 main effects or interactions warrant mention and discussion given the relatively small groups and high inter-subject variability of some data. This can also be considered in a Discussion of limitations of this research.

Gandevia & Heroux (2019, J Physiol) have outlined guidelines for statistical analysis and interpretation for Journal of Physiology. They suggested caution against interpreting p values above 0.05. Therefore, in line with the journal guidelines, we refrained from interpreting these values or highlighting these p values to ensure we did not implicate their meaning in our discussion.

We have added to the discussion a statement on this limitation:
(P.24, L.560-562) "*No other longitudinal changes were observed, which may be the result of the small sample size and the large variability between participants.*"

However, following a suggestion of the editors and other reviewer, we have added effect sizes and confidence intervals to not solely relying on the P values in the results.

We also discussed the yield of motor units and its impact on our results:
(P.25-26, L.609-619): *"Thus, PSTH-derived metrics may be considered as a biomarker when assessing patients' control of balance. However, the application of such approaches is currently limited to high quality recordings capable of extracting multiple motor unit firings in patients. For example, it is noteworthy that our regression analysis only included individuals with motor units extracted in both visits, with three individuals in the stroke group excluded from all motor unit analyses due to the inability to extract any motor units. The absence of extracted motor units may be explained by physiological factors, such as low muscle activation or reduced neural drive, but also poor signal quality, which are particularly present after stroke. Therefore, to extend the applicability of motor unit-based clinical analyses, future work must continue to improve HD-EMG sensors, extend investigations to intramuscular EMG, and enhance decomposition techniques to increase motor unit yields across wider populations."*

Gandevia, Simon, and Martin Héroux. "Statistics and Spin: It's Time to Improve." The Journal of Physiology [England], vol. 597, no. 16, August 2019, pp. 4411–12, https://doi.org/10.1113/JP278335.

8. Figure 3: What exactly is meant by "Area under the curve between -0.4s to 0s relative to the peak." in relation to the ordinate scales for PSTH amplitude in A and B? How was the time from -0.4s to 0s determined to be the appropriate time-period to evaluate?
We specify the approach to compute the EMG and PSTH activation just prior to the COP peak (computing the area of 400ms before the event) in the Methods.
(P.9, L.230-234) "*To quantify the PSTH and EMG activation amplitude during COP peaks in the four directions, the area under the curve from -400ms to 0ms relative to the time of the COP peak was computed. This time was selected to quantify the activity that resulted in the change in direction and the resulting COP peak, as done in previous works (Zaback et al., 2023; Zaback & Thompson, 2026).*"

We have updated the caption to specify that the time is relative to the peak in COP.
(P.16, L.386-387) "*Area under the curve between -0.4s to 0s relative to the peak in COP.* "

What exactly are the plotted numbers of the ordinate scales for PSTH amplitude in A and B as they are quite different but have the same units (pulses/peak).

Thank you for pointing out this discrepancy. All values should be represented as pulses or μV per peak. After reviewing the data, a scaling factor was missed on the area calculation for the PSTH that has since been corrected. This has been adjusted in Figure 3 (P.16; L.383):

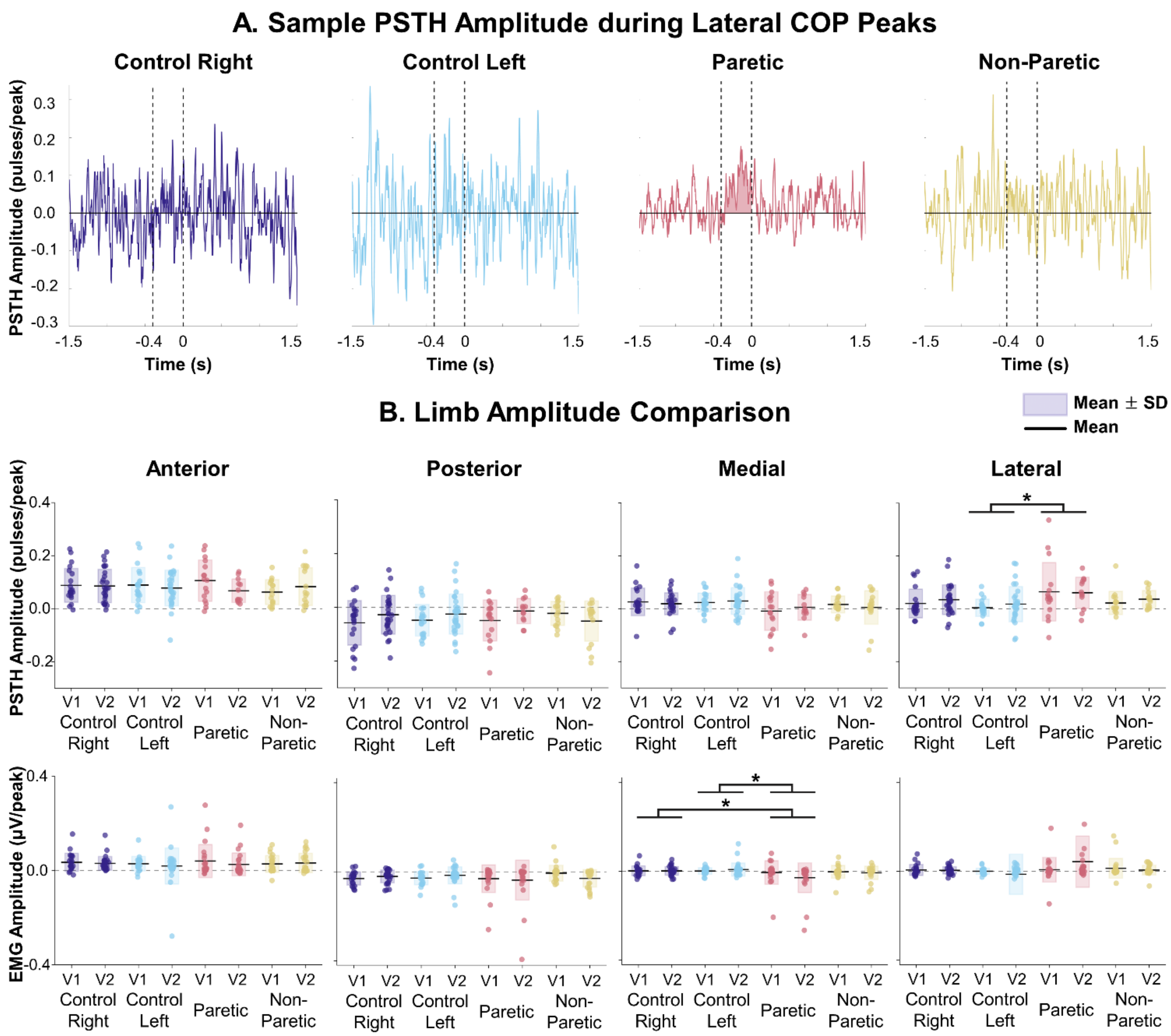

Also, are these data in a and B from eyes open or closed conditions?

These data are from eyes closed, visit 2 for both the control and stroke participant sample data. This has been reported in the caption:

(P.16, L.384-386) "*Figure 3: Activation Amplitude. A) Sample peristimulus time histogram (PSTH) amplitude from one control and one stroke participant during lateral center of pressure (COP) peaks in the eyes closed task during visit 2.*"

This has also been specified for Figure 6.
(P.21, L.473-476) "***Figure 6: Lag between* bilateral *soleus activities****. A) Bilateral smoothed peristimulus time histogram (PSTH) time-locked to anterior center of pressure (COP) peaks for a sample control and stroke participant from visit 1 and visit 2 during the eyes closed task. The graphical display of time lag is depicted, and the black line indicates the time of COP peak.*"

9. Under "Directional tuning", the variability of mean angles among subjects in both groups for each leg is very high for both PSTH and EMG data. For example, control right and left limbs for PSTH had mean +/- SDs of 0.79 o +/- 40.62o and 6.54 o +/- 37.56 o. Indeed, according to Fig. 4B absolute differences of mean angles of the two legs exceeded 90o in several control subjects. How similar (reliable) were the mean angles for each leg and the absolute differences between legs in visit 1 and visit 2 in controls? Is it reasonable to assume that the absolute difference between angles for the two legs signifies a real difference? This issue should be discussed as a limitation in the interpretation of directional tuning data.
The reviewer is correct that some healthy participants showed substantial variability on the mean angle of the tuning curve. From a methodological point of view, variability can result from a limited number of COP peaks used to compute the tuning curves, shifts in COP that do not return to center, and noisy data. To address the limitations, we ran a simulation to determine how the number of peaks in COP affects the directional tuning curves. The outcome of this preliminary step enabled us to identify a relative threshold required to achieve accurate tuning curves. Additionally, we used an adaptive threshold computed over sliding windows of 5 sec to ensure that we did not misidentify peaks due to shifts in COP as in a previous study of Zaback and Thompson (2026; J Appl Physiol).

Methods:
(P.9, L.212-219) "*In neurologically intact individuals, the standard deviation of the high-pass filtered (second order, 0.1 Hz cut-off dual-pass Butterworth) COPs was used to set an individualized threshold for identifying prominent peaks. However, as individuals post-stroke often display large balance adjustments that may skew this threshold, standard deviation was calculated across segmented high-pass filtered COP data (window size = 5s, step size = 1.25s, total of 24 segments/30s trial), and the lower 25th percentile of these measures of variability was used to set an optimal peak detection threshold that would be non-paretic by the presence of aberrant fluctuations.*"

While the shorter trial duration in our study–compared to that of Zaback and Thompson, due to patients' limited ability to sustain prolonged standing–may introduce variability, the alignment of the mean angles of the soleus tuning curves in healthy controls with their findings suggests that the method remains robust even with shorter trials.

From a physiological perspective, the higher variability observed here compared to the previous study of Zaback and Thompson may be due to the demographic of our control population, which was age-matched with the patients post stroke. Aging can impact postural and balance control, which may have turned here for some participants in a difference in mean directional angle

between legs, due to, e.g., increased coactivation. We have addressed this limitation in the discussion.

(P.23; L.517-528): "*It is noteworthy that the mean angles of the tuning curve observed here exhibited higher variability than those measured by (Zaback & Thompson, 2026) (SD: 37-47° vs. 17.7°). This difference may result from differences in demographics for the control group (8 males/females, mean age: 61.2±14.7 years here vs. 10 males; mean age: 29.9±7.2 years in Zaback and Thompson, 2026), as older individuals often experience impairments in balance control (Granacher et al., 2011; Matsuda et al., 2025). Another factor explaining this variability could be the duration of trials used to construct the tuning curves, which was limited here – compared to our previous study in neurologically intact individuals – due to the inability of our patients to maintain postures for long periods of time. However, the mean angles of control and non-paretic limbs align with the previous study (Zaback & Thompson, 2026), suggesting the robustness of the method with shorter trials.*"

Overall, we are confident that there is a clear difference in the directional tuning of the paretic limb compared to the other three limbs, which is the main outcome of this analysis.

Zaback M & Thompson CK. (2026). Independent directional tuning of the human triceps surae muscles during standing postural control. J Appl Physiol (1985).

Finally, EMG amplitude is not normalized, yet across-group comparisons are made. Of course, normalization is difficult in stroke patients but the lack of normalization should be discussed in a Discussion section on limitations.
It is true that normalization of EMG signals during balance or dynamic tasks is challenging. However, this step was not necessary here. First, the directional tuning analysis is computed from the relative activation of the same muscle across the 72 angles. This can be done over normalized or non-normalized data, with both giving the same result. Second, the EMG amplitude was estimated from an entire grid of 64 electrodes separated by a distance of 8mm. While normalization is needed to limit the impact of signal cancellation on the estimation of muscle activation with bipolar electrodes (Keenan et al., 2005, J Appl Physiol), this is less important for grids of electrodes. Indeed, the higher spatial sampling of the signal decreases the impact of signal cancellation on the estimation of EMG amplitude. As such, several studies have used raw EMG amplitudes estimated from the grid of electrodes to quantify changes in muscle activation (e.g., Vila-Chã et al., 2010, J Appl Physiol). We chose to follow this approach with the motivation to not add unnecessary fatigue with maximal contraction to our participants.

Keenan KG, Farina D, Maluf KS, Merletti R, Enoka RM. Influence of amplitude cancellation on the simulated surface electromyogram. *J Appl Physiol (1985)*. 2005;98(1):120-131.

Vila-Chã C, Falla D, Farina D. Motor unit behavior during submaximal contractions following six weeks of either endurance or strength training. *J Appl Physiol (1985)*. 2010;109(5):1455-1466.

10. Figure 4A: The control left and unaffected leg tuning curves should be plotted in brighter colors as they are difficult to see.
The figures have been updated to make the plots bigger and focus the visualization on the difference between visits (P.18; L.414):

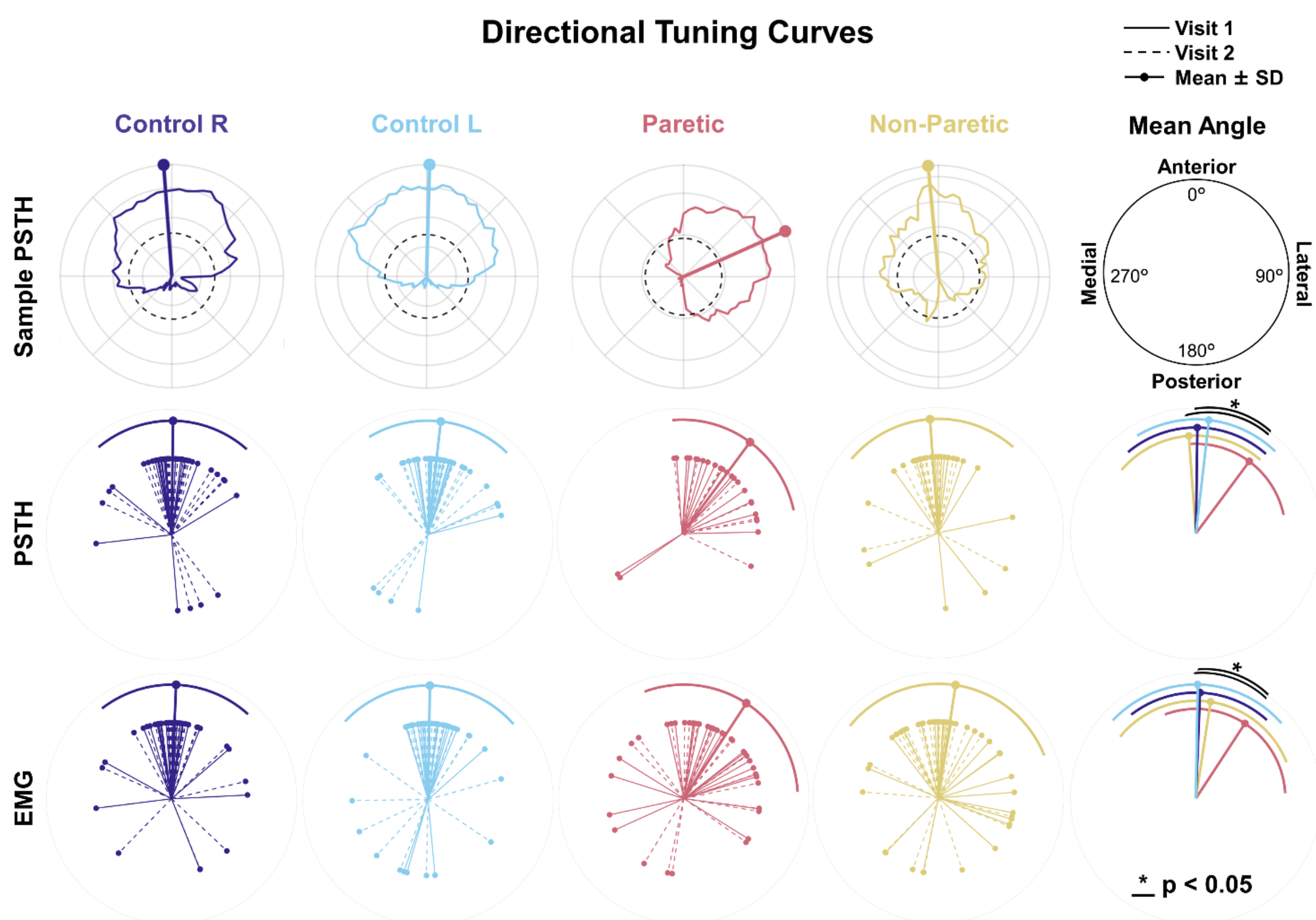

11. Figure 5: The legend next to C is, I think, actually the legend for D.
This has been adjusted.

Also, are the examples in A and B from eyes open or closed conditions and from visit 1 or 2?
Both examples are from the eyes closed condition.

The caption has been updated accordingly:
(P.19, L.439-442) "Figure 5: Contralateral-Triggered Activation. *A) control right (purple) and left (blue) and B) stroke participant paretic (red) and non-paretic (yellow) ipsilateral- and contralateral-triggered peristimulus time histogram (PSTH) waveforms during standing with eyes closed (visit 2 for the control and visit 1 for the stroke participant).* "

12. Under "Cross correlation of motor unit...", it is stated: "Controls demonstrated greater correlation coefficients (0.79{plus minus}0.19) compared to the stroke group (0.68{plus minus}0.17)." However, no p-value for the statistical comparison of the correlation coefficients is provided, thus it is not clear that the coefficients were statistically larger in controls.

We have added the p value in:
(P.19; L463-465) "*Controls demonstrated greater correlation coefficients (0.79±0.19) compared to the stroke group (0.68±0.17, **P=0.005**, d=0.381, 95% CI: [-0.017, 0.779]).*"

13. Under "Forward regression ...", it is stated: "Data was pooled across tasks as task effects were minimal." However, this pooling of data would provide multiple data points for each subject whereas such regressions would normally be performed using a single data point for each subject. Using two data points from each subject would violate statistical assumptions of independence for each data point. Figure 7 has 22 data points, indicating there were two from each of the 11 stroke subjects who had PSTH neuromuscular data. It would be more appropriate to plot the data with different symbols for eyes-open vs closed conditions and perform separate regression for those conditions. Also, it should be specified whether BBS and neuromuscular data was from visit 1 or visit 2 was used in Fig. 7. Relatedly, it would be interesting to provide a similar graph and regression coefficients for visit 2 given the improvement of BBS scores in visit 2.
Thank you for this comment, which was also pointed out by the other reviewer. Following your advice, we have pooled the data per participant and ran two analyses. First, we have performed a linear regression between delta lag and delta Berg Balance scale score between visits, showing a negative significant correlation between both variables ($r = -0.94$; $P < 0.001$). Second, as you suggested, we ran a repeated measures correlation to further confirm this result ($r = -0.78$; $P = 0.002$). The results have been updated to report these analyses:

(P. 21-22; L.479-487) "*Only PSTH absolute lag demonstrated a significant longitudinal change for stroke participants. Therefore, the relationship between the change in PSTH absolute lag and the change in functional balance score (i.e., BBS score) for stroke participants was assessed via linear regressions. Data was averaged across tasks per participant as task effect was absent. A linear regression between the changes in lag between visits and the change in BBS score between visits revealed a significant negative correlation (r=-0.94, P<0.001). This was further confirmed by a repeated measures correlation analysis with a significant negative correlation between both variables (r = -0.78; p = 0.002). Thus, an increase in BBS score was accompanied by a decrease in lag (or an increase in synchronization).*"

Discussion

Overall, this section is clearly written and provides reasonable interpretations of the findings. However, as mentioned above, there are some issues that should be addressed in a discussion section on limitations related to interpretations of these data.
Please see our responses to your comments above.

Formatting

The following formatting updates have also been made:

- Changing the citation style and moving all citations before the punctuation.

- Added an additional information section
- Added a graphical abstract

Re: JP-RP-2026-290882R1 "Disrupted Bilateral Coordination of Soleus Motor Units during Early Subacute Stroke Rehabilitation" by Jackson T Levine, Xin S Yu, Alyssa Jones, Rebecca Munoz, Martin Zaback, Christopher K Thompson, Dario Farina, Simon Avrillon, and José L. Pons

Dear Dr Levine,

Thank you for submitting your manuscript to The Journal of Physiology. It has been assessed by a Reviewing Editor and by 2 expert referees and we are pleased to tell you that it is potentially acceptable for publication following satisfactory major revision.

Please advise your co-authors of this decision as soon as possible.

The referee reports are copied at the end of this email.

Please address all the points raised and incorporate all requested revisions or explain in your Response to Referees why a change has not been made. We hope you will find the comments helpful and that you will be able to return your revised manuscript within 2 months. If your article is NOT for a Special Issue, you may have 9 months to revise. If you require an extension, please contact journal staff: jp@physoc.org. Please note that this letter does not constitute a guarantee for acceptance of your revised manuscript.

Your revised manuscript should be submitted online using the link in your Author Tasks: Link Not Available. This link is accessible via your account as Corresponding Author; it is not available to your co-authors. If this presents a problem, please contact journal staff (jp@physoc.org). Image files from the previous version are retained on the system. Please ensure you replace or remove any files that are being revised.

If you do not wish to submit a revised version of your manuscript, you must inform our journal staff (jp@physoc.org) or reply to this email to request withdrawal. Please note that a manuscript must be formally withdrawn from the peer review process at one journal before it may be submitted to another journal.

TRANSPARENT PEER REVIEW POLICY: To improve the transparency of its peer review process, The Journal of Physiology publishes online as supporting information the peer review history of all articles accepted for publication. Readers will have access to decision letters, including Editors' comments and referee reports, for each version of the manuscript, as well as any author responses to peer review comments. Referees can decide whether or not they wish to be named on the peer review history document.

ABSTRACT FIGURES: Authors are expected to use The Journal's premium BioRender account to create/redraw their Abstract Figures. Information on how to access this account is here: https://physoc.onlinelibrary.wiley.com/journal/14697793/biorender-access.

This will enable Authors to create and download high-resolution figures. If authors have used the free BioRender service, they can use the instructions provided in the link above to download a high-resolution version suitable for publication.

The link provided should only be used for the purposes of this submission. Authors will be charged for figures created on this account if they are not related to this manuscript submission.

LANGUAGE EDITING AND SUPPORT FOR PUBLICATION: If you would like help with English language editing, or other article preparation support, Wiley Editing Services offers expert help, including English Language Editing, as well as translation, manuscript formatting, and figure formatting at www.wileyauthors.com/eeo/preparation. You can also find resources for Preparing Your Article for general guidance about writing and preparing your manuscript at www.wileyauthors.com/eeo/prepresources.

RIGOUR AND REPRODUCIBILITY: We recommend authors consult The Journal's Rigour and Reproducibility requirements to ensure that their manuscript is suitable for publication: https://physoc.onlinelibrary.wiley.com/pb-assets/hub-assets/physoc/documents/TJP-Rigour-and-Reproducibility-Requirements.pdf

REVISION CHECKLIST:

Check that your Methods section conforms to journal policy: https://jp.msubmit.net/cgi-bin/main.plex?form_type=display_requirements#methods.

Check that data presented conforms to the statistics policy: https://jp.msubmit.net/cgi-bin/main.plex?form_type=display_requirements#statistics.

Upload a full Response to Referees file. To create your 'Response to Referees': copy all the reports, including any comments from the Senior and Reviewing Editors, into a Microsoft Word, or similar, file and respond to each point, using font or background colour to distinguish comments and responses and upload as the required file type.

Please upload two versions of your manuscript text: one with all relevant changes highlighted and one clean version with no changes tracked. The manuscript file should include all tables and figure legends, but each figure/graph should be uploaded as separate, high-resolution files.

You may also upload:

- 'Potential Cover Art' for consideration as the issue's cover image
- Appropriate Supporting Information (Video, audio or data set: see https://jp.msubmit.net/cgi-bin/main.plex?form_type=display_requirements#supp).

We look forward to receiving your revised submission.

If you have any queries, please reply to this email and we will be pleased to advise.

Yours sincerely,

Richard Carson
Senior Editor
The Journal of Physiology

EDITOR COMMENTS

Thank you for your response, the depth of which is acknowledged by both reviewers. However, as you will see form their comments, several outstanding points remain to be fully addressed. Please address all points relating to the possible influence of physiotherapy assistance, and the limitations of not normalising EMG.

Senior Editor:

As highlighted in comments provided by Referee #1, there is a requirement for a more complete integration and nuanced interpretation of the effect size statistics. While the inclusion of these measures is a positive step, there is more that might/should be done in terms of utilising the information they provide.

REFEREE COMMENTS

Referee #1:

The authors have made substantial improvements to the paper. I have one concern remaining and a few minor comments.

Major: Influence of Physiotherapy Assistance

Original point 6. I am not at all satisfied with a strictly statistical approach to mitigating the impact of physiotherapist assistance on the outcomes, especially with a small sample size. I would like to SEE the COP data a) for participants in which a) the assistance was 0-2 vs. 3-5 and b) for participants in which the amount of assistance did not change between visits 1 and 2 and c) for those where the assistance did change, how did the COP data change?

In response to comment 8, the plot of assistance vs. BBS, there are 35 data points (14 participants, 2 visits) Why not 28 data points? Probably because some had a different assist level with eyes closed than eyes open. While I think the regression line is not helpful because the data points are not independent, the graph illustrating that only 5 data points of the 35 had assistance levels of 3-5 is very helpful, maybe more so than the averages reported in lines 572-574, which I did appreciate nonetheless.

I would recommend that in the first paragraph of results, the authors state exactly how many participants needed assistance (3-5) on visit 1 and on visit 2. My guess is that 5 of 14 needed assistance on visit 1 and none on visit 2.

Minor:

The graphical abstract has some typos - laterally, Berg Balance Scale

Line 151 - how does a patient with a BBS of 5 engage in "high intensity gait training"? There must have been rapid

improvement!

Fig 2 legend - COP displacement

Line 450, should the final description refer to right column?

Line 488 - Data were (not was)

Original point 8. The reviewer is well aware of Gandevia's position on statistics. My point was not that you should report 'trends' but that you were likely underpowered and that should be acknowledged. Furthermore, my recommendation of reporting effect sizes is in line with Gandevia's recommendations. The fact that you report a p=0.113 with a d=0.448 (a non-significant finding with a medium effect size) proves my point. Yet, you fail to comment on the cohen d...

Referee #2:

The authors have satisfactorily addressed all but one of my comments, which was related to the lack of EMG normalization. The authors pointed out that normalization of the EMG signal is not necessary for the directional tuning analysis, with which I agree. However, I disagree that normalization is not necessary for comparison of EMG amplitudes across groups and sessions when using grid electrodes. The authors suggest that normalization is needed primarily to limit the impact of signal cancellation when using bipolar electrodes whereas this is less of a problem with electrode grids. However, normalization is generally considered necessary for across-subject/group comparisons due to inter-subject differences in skin and subcutaneous tissue thickness and electrical properties of these tissues. It is well-known that session-to-session absolute EMG amplitudes for equivalent contractions have low reliability without normalization. Thus, the lack of normalization for comparison of EMG amplitudes should be discussed as a limitation in the Discussion section.

END OF COMMENTS

EDITOR COMMENTS

Thank you for your response, the depth of which is acknowledged by both reviewers. However, as you will see form their comments, several outstanding points remain to be fully addressed. Please address all points relating to the possible influence of physiotherapy assistance, and the limitations of not normalising EMG.

We thank the editors and the reviewers for their comments on our manuscript. We especially appreciate the feedback on the need for more transparency on the physiotherapy assistance and how it can impact our outcomes. We fully agree with reviewer #1 on their point, and hope that our revision will satisfy them.

We also understand the comment on EMG normalisation from reviewer #2 and address their concern in the new version of our manuscript.

Overall, we believe that this new version will enable readers to have a clearer reading of our results in regard to the specific conditions in which the data were gathered.

Senior Editor:

As highlighted in comments provided by Referee #1, there is a requirement for a more complete integration and nuanced interpretation of the effect size statistics. While the inclusion of these measures is a positive step, there is more that might/should be done in terms of utilising the information they provide.

We have revised the results to take into consideration the moderate effect sizes associated with p values close to 0.05. Please find our responses under each reviewer's comments.

REFEREE COMMENTS

Referee #1:

The authors have made substantial improvements to the paper. I have one concern remaining and a few minor comments.

Thanks for the positive feedback on our revision. Please find below our edits related to physiotherapy assistance and the better consideration of effect sizes in our results.

Major: Influence of Physiotherapy Assistance

Original point 6. I am not at all satisfied with a strictly statistical approach to mitigating the impact of physiotherapist assistance on the outcomes, especially with a small sample size. I would like to SEE the COP data a) for participants in which a) the assistance was 0-2 vs. 3-5 and b) for participants in which the amount of assistance did not change between visits 1 and 2 and c) for those where the assistance did change, how did the COP data change?

Please find below the answers to your questions. Note that we have added this information to both the results and the discussion to clarify the level of assistance of the physical therapists and highlight how this assistance can impact the outcomes of the study. For the sake of clarity, we report here the COP displacement in both anteroposterior and mediolateral directions rather than both the displacement and speed.

a) **Week 1.** On average, the COP displacement was 13.3±10.6mm in the AP direction and 14.6±11.8mm in the ML direction for patients with a score between 0 and 2. On average, the COP displacement was 29.9±6.0mm in the AP direction and 30.5±12.1mm in the ML direction for patients with a score between 3 and 5.

   Week 2. On average, the COP displacement was 8.6±3.7mm in the AP direction and 10.5±9.6mm in the ML direction for patients with a score between 0 and 2. On average, the COP displacement was 14.8±7.0mm in the AP direction and 18.5±5.3mm in the ML direction for patients with a score between 3 and 5.

   Therefore, both subgroups of patients improved between the two sessions, and patients with a score between 3 and 5 constantly had a higher displacement.

b) On average, the COP displacement decreased by 3.8±9.1mm in the AP direction and by 5.2±11.2mm in the ML direction in patients with no change in level of assistance.
c) On average, the COP displacement decreased by 6.0±11.8mm in the AP direction and by 5.8±15.6mm in the ML direction in patients with a change in level of assistance.

   This data shows that both groups improved on average, despite a change in assistance level for half of the patients between visits.

We added a sentence in the methods to be clear about the potential assistance provided by the therapist:

(P.8; L.160) *"All participants wore a harness for safety that did not provide support to the participant during standing, and a physical therapist provided assistance as necessary to prevent patients from falling."*

We also added a comment in the discussion to highlight how this assistance can impact the reading of our results:

(P.24; L.560) *"It is noteworthy that the physical therapist assistance may have also impacted these outcomes, as even short touch assistance from the therapist likely reduced COP displacement and speed around COP peaks in some directions. However, the touch assistance was only performed to prevent a fall of the patient, and the three patients with score of 3 to 5 still exhibited higher COP displacement than patients with a score of 0 to 2 (e.g., during week 1: 29.9±6.0mm and 30.5±12.1mm in AP and ML directions vs. 13.3±10.6mm and 14.6±11.8mm respectively). Moreover, the level of assistance did not change over time (average change: -0.43±0.63 points out of 5, P=0.251), and both groups of patients reduced their COP displacement between visits"*

In response to comment 8, the plot of assistance vs. BBS, there are 35 data points (14 participants, 2 visits) Why not 28 data points? Probably because some had a different assist level with eyes closed than eyes open. While I think the regression line is not helpful because the data points are not independent, the graph illustrating that only 5 data points of the 35 had assistance levels of 3-5 is very helpful, maybe more so than the averages reported in lines 572-574, which I did appreciate nonetheless

Yes, the level of assistance was different between eyes closed and eyes open for some patients. On average, the level of assistance was 1.8 (range: 1-5) and 1.3 (range: 0-5) for eyes closed and eyes open in week 1. It was 1.3 (range: 0-4) and 0.9 (range: 0-4) for eyes closed and eyes open in week 2.

I would recommend that in the first paragraph of results, the authors state exactly how many participants needed assistance (3-5) on visit 1 and on visit 2. My guess is that 5 of 14 needed assistance on visit 1 and none on visit 2.

We clarified the level of assistance at the beginning of the result section: (P.14; L.349): *"The score of assistance provided by the therapist during the standing task was on average 1.5±1.3 (range: 0-5) during the first session and 1.1±1.2 (range: 0-4) during the second session. Three and two patients required assistance (score 3 to 5) during eyes closed and eyes open in week 1, respectively. Two patients required assistance (score 3 to 5) during eyes closed and eyes open in week 2. Six patients required less assistance during the second session while standing eyes closed (-0.5±0.7). Six patients required*

less assistance during the second session while standing eyes open (-0.4±0.4), and one patient required more assistance during the second session, with a score moving from 1 to 2."

Minor:

The graphical abstract has some typos - laterally, Berg Balance Scale

Typos were removed.

Line 151 - how does a patient with a BBS of 5 engage in "high intensity gait training"? There must have been rapid improvement!

Each session of gait training consistently began with a 5- to 10-minute warm-up, after which the intensity was gradually increased to reach 70–85% of the theoretical maximum heart rate and a score of 14 to 17 on the Borg Rating of Perceived Exertion scale. Patients took breaks as often as necessary during the sessions. At the start of rehabilitation and whenever required, the physiotherapist fitted the patient with a harness to provide support and assistance during walking, thereby improving gait mechanics and promoting continuous walking without collapse of the trunk and/or limbs. As implied by the reviewer, the patient with a BBS of 5 was likely supported during the entire session, with assistance from therapists to move their legs over the treadmill during the swing phases. To avoid any confusion, we removed *"(with a focus on high-intensity gait training)"* from the description of the therapy.

Fig 2 legend - COP displacement

Changed as suggested.

Line 450, should the final description refer to right column?

You are right, and this was changed:

(P20; L455) "*Figure 5: Contralateral-Triggered Activation. A) Control right (purple) and left (blue) and B) post-stroke participant paretic (red) and non-paretic (yellow) ipsilateral- and contralateral-triggered peristimulus time histogram (PSTH) waveforms during standing with eyes closed (visit 2 for the control and visit 1 for the stroke participant). The smoothed cumulative spike train (CST) is displayed with the peaks shown with circles. The color code is based on the limb from which the peaks were detected. As a guide to read these figures, for the top row of Panel A, the left column is the CST of the control right limb. The*

middle column is the ipsilateral activation of the right limb during right limb peaks in CST. The ***right column*** *is the contralateral activation of the left limb during right limb peaks in CST. C) Difference between the amplitude of the contralateral (contra) and ipsilateral (ipsi) triggered activation amplitudes. * P<0.05. R = Right. L = Left. Par = Paretic. Non = Non-Paretic. V = Visit. pps = Pulses Per Second*."

Line 488 - Data were (not was)

Changed as suggested.

Original point 8. The reviewer is well aware of Gandevia's position on statistics. My point was not that you should report 'trends' but that you were likely underpowered and that should be acknowledged. Furthermore, my recommendation of reporting effect sizes is in line with Gandevia's recommendations. The fact that you report a p=0.113 with a d=0.448 (a non-significant finding with a medium effect size) proves my point. Yet, you fail to comment on the cohen d...

We apologise for the misunderstanding and now acknowledge these differences in the results.

(P. 16; L. 388): *"For EMG in the medial direction, there was an effect of limb (P=0.018), with the amplitude in the paretic limb (-0.018±0.061 µV/peak) lower than in the right limb of controls (0.002±0.020 µV/peak, P=0.042, d=0.557, 95% CI: [0.160, 0.953]) and left limb of controls (0.005±0.024 µV/peak, P=0.026, d=0.590, 95% CI: [0.185, 0.996]), and* ***a moderate difference with the non the non-paretic limb, albeit non-significant with our sample size*** *(-0.005±0.029 µV/peak, P=0.113, d=0.448, 95% CI: [0.037, 0.860]."*

(P. 18; L. 418): *"****Despite a moderate effect size, there was no statistically significant*** *difference with the left control limb (6.54±37.56°, P=0.012, d=0.770, 95% CI: [0.294, 1.247]). For EMG amplitudes, the mean angle of the tuning curve was significantly more lateral for the paretic limb (33.24±53.41°) than for the right control limb (2.27±39.90°, P=0.005, d=0.662, 95% CI: [0.267, 1.056]) and the left control limb (0.93±47.75°, P=0.008, d=0.640, 95% CI: [0.235, 1.044]).* ***There was a moderate difference between the paretic and the non-paretic limb, albeit non-significant with our sample size (8.67±59.53°, P=0.093, d=0.434, 95% CI: [0.025, 0.844]).****"*

We acknowledged that our study was likely underpowered in the discussion: (P. 27; L.629): *"Alternatively, EMG-derived parameters that do not rely on decomposed*

signals could also become biomarkers, though our study was likely underpowered to demonstrate this."

Referee #2:

The authors have satisfactorily addressed all but one of my comments, which was related to the lack of EMG normalization. The authors pointed out that normalization of the EMG signal is not necessary for the directional tuning analysis, with which I agree. However, I disagree that normalization is not necessary for comparison of EMG amplitudes across groups and sessions when using grid electrodes. The authors suggest that normalization is needed primarily to limit the impact of signal cancellation when using bipolar electrodes whereas this is less of a problem with electrode grids. However, normalization is generally considered necessary for across-subject/group comparisons due to inter-subject differences in skin and subcutaneous tissue thickness and electrical properties of these tissues. It is well-known that session-to-session absolute EMG amplitudes for equivalent contractions have low reliability without normalization. Thus, the lack of normalization for comparison of EMG amplitudes should be discussed as a limitation in the Discussion section.

We agree with the reviewer on their comment on the importance of normalisation to mitigate the effect of signal cancellation and allow the comparison of values between participants. Note that in our study, we refrained from interpreting such differences and focused our analyses on the difference in EMG amplitude in windows triggered by peaks of activity of the soleus in the contralateral side vs. its own peaks of activity. Therefore, we compare the relative difference in EMG amplitude of the same muscle with the same grid in two different time windows. We have now added a paragraph in the discussion to clarify this analysis:

(P. 25; L.576): *"This result was confirmed by also evaluating EMG amplitude of soleus muscles during peaks in activity in the contralateral limb. There was a lower activity in the soleus of the paretic limb during peaks of activity of the other soleus compared to its own peaks of activity. In controls, there were no differences in amplitude when triggered by the activity in the contralateral side or by its own peaks of activity. Note that due to the lack of normalization of the EMG amplitude, we cannot interpret EMG amplitudes as an indicator of muscle activation and cannot compare the raw values between participants and sessions. Here, we only interpret the relative differences in amplitude within the same muscle using the entire grid of electrodes."*

2nd Revision - Editorial Decision **25-Aug-2026**

Dear Dr Levine,

Re: JP-RP-2026-290882R2 "Disrupted Bilateral Coordination of Soleus Motor Units during Early Subacute Stroke Rehabilitation" by Jackson T Levine, Xin S Yu, Alyssa Jones, Rebecca Munoz, Martin Zaback, Christopher K Thompson, Dario Farina, Simon Avrillon, and José L. Pons

We are pleased to tell you that your paper has been accepted for publication in The Journal of Physiology.
- Authors are required to fully disclose the use of any Generative AI (GAI) tools used in the preparation of their manuscript. This disclosure should also be included as a statement alongside the Additional Information and Data Availability statements towards the end of the article under the heading 'Generative AI Statement', and include the name(s), model and version of AI technology used, and exact details on the purpose for, and methods of, its use.

If no AI tool has been used, this should also be specifically stated within the manuscript.

The last Word (or similar) version of the manuscript provided will be used by the Production Editor to prepare your proof. When this is ready you will receive an email containing a link to Wiley's Online Proofing System. The proof should be thoroughly checked and corrected as promptly as possible.

Authors should note that it is too late at this point to offer corrections prior to proofreading. Major corrections at proof stage, such as changes to figures, will be referred to the Editors for approval before they can be incorporated. Only minor changes, such as to style and consistency, should be made at proof stage. Changes that need to be made after proof stage will usually require a formal correction notice.

All queries at proof stage should be sent to: TJP@wiley.com.

Following copyediting and typesetting the accepted version will be published online.

Yours sincerely,

Richard Carson
Senior Editor
The Journal of Physiology

IMPORTANT POINTS TO NOTE FOLLOWING ACCEPTANCE OF YOUR PAPER:

• IMPORTANT NOTICE ABOUT OPEN ACCESS: To assist authors whose funding agencies mandate immediate public access to published research findings, The Journal of Physiology allows authors to pay an Open Access (OA) fee to have their papers made freely available immediately on publication.

The Corresponding Author will receive an email from Wiley with details on how to register or log in to Wiley Authors where you will be able to place an order.

• You can check if your funder or institution has a Wiley Open Access Account here:
https://authors.wiley.com/author-resources/Journal-Authors/open-access/author-compliance-tool.html

MEMBER DISCOUNT: Corresponding authors who are Members of the Physiological Society are eligible for a 20% discount on the cost of the Article Processing Charge (APC). To claim this discount, the Corresponding Author will need to log in to the Physiological Society Membership portal and retrieve the discount code (https://www.physoc.org/journals-and-media/journals/journal-discount-for-members/). This code should then be quoted in the billing section of Wiley Authors when selecting the open access option.

• TRANSPARENT PEER REVIEW POLICY: To improve the transparency of its peer review process, The Journal of Physiology publishes online as supporting information the peer review history of all articles accepted for publication. Readers will have access to decision letters, including Editors' comments and referee reports, for each version of the manuscript as well as any author responses to peer review comments. Referees can decide whether or not they wish to be named on the peer review history document.

• You can help your research get the attention it deserves! Check out Wiley's free Promotion Guide for best-practice recommendations for promoting your work at: www.wileyauthors.com/eeo/guide. You can learn more about Wiley Editing Services which offers professional video, design, and writing services to create shareable video abstracts, infographics, conference posters, lay summaries, and research news stories for your research at: www.wileyauthors.com/eeo/promotion.

• If you would like to receive our 'Research Roundup', a monthly newsletter highlighting the cutting-edge research published in The Physiological Society's family of journals (The Journal of Physiology, Experimental Physiology, Physiological Reports, The Journal of Nutritional Physiology and The Journal of Precision Medicine: Health and Disease), please click this link, fill in your name and email address and select 'Research Roundup':
https://www.physoc.org/journals-and-media/membernews

EDITOR COMMENTS

Reviewing Editor:

Thank you for addressing all comments.

Please correct any typos at proofing stage.

REFEREE COMMENTS

Referee #1:

I thank the authors for addressing my remaining concerns. Other than a typo on line 385, I have no further comments.

line 384-385 "and with a moderate difference with the non the non-paretic limb, albeit non-significant with our sample size"

Referee #2:

The authors have responded appropriately to my comment, resulting in an improved manuscript.
